# Mapping the Disrupted Connectome in Spinocerebellar Ataxia Type 3: A Network‐Based Statistics Study Identifying Novel Therapeutic Targets for Neuromodulation

**DOI:** 10.1002/cns.71016

**Published:** 2026-07-06

**Authors:** Lan Ou, Chaoyang Zhou, Xingang Wang, Linfeng Shi, Peiling Ou, Chaodong Xiang, Hui Chen, Xi Chen, Bijia Wang, Wei Chen, Jian Wang, Zhiliang Long, Chen Liu

**Affiliations:** ^1^ 7T Magnetic Resonance Imaging Translational Medical Center, Department of Radiology Southwest Hospital, Army Medical University (Third Military Medical University) Chongqing China; ^2^ Department of Neurology Southwest Hospital, Army Medical University (Third Military Medical University) Chongqing China; ^3^ The Southwest University Chongqing China

**Keywords:** functional connectivity, network‐based statistics, spinocerebellar ataxia type 3, structural connectivity, transcranial magnetic stimulation

## Abstract

**Aims:**

Spinocerebellar ataxia type 3 (SCA3) is characterized by progressive neurodegeneration. This study aimed to map alterations in structural and functional connectivity (SC and FC) in SCA3 using network‐based statistics (NBS) and to explore the potential of transcranial magnetic stimulation (TMS) for modulating these aberrant networks and identifying novel therapeutic targets.

**Methods:**

NBS was used to compare SC and FC between a large cohort of 117 SCA3 patients and 163 healthy controls (HCs). Partial correlation analysis examined the associations between altered connectivity and clinical variables (CAG repeat length, disease duration, and clinical scale scores). Additionally, paired *t*‐tests were used to evaluate longitudinal brain connectivity changes in 58 patients before and after TMS intervention to assess therapeutic effects.

**Results:**

SCA3 patients exhibited a distinct disconnection–compensation pattern, characterized by significantly reduced SC and elevated FC within the subcortical (SUB), sensorimotor (SMN), and dorsal attention (DAN) networks. SC between the frontoparietal network (FPN) and SUB was abnormally increased and positively correlated with both disease duration and CAG repeat length, whereas intra‐SUB SC was negatively correlated with disease duration. Following TMS intervention, SC strength in two pathways that were abnormally enhanced at baseline was significantly attenuated.

**Conclusion:**

Our findings reveal extensive connectome reorganization in SCA3. The correlations between SC abnormalities and clinical metrics, coupled with the modulatory effects of TMS, suggest that network‐based metrics can serve as biomarkers and guide the development of personalized neuromodulation strategies for SCA3.

**Trial Registration:** (ChiCTR) 1800019901, 2000039434, 2500095738.

## Graphical abstract

This study identifies a dual pattern of structural and functional connectome disruption in SCA3 that correlates with clinical severity. Importantly, these abnormal structural connectivities are modulated by TMS, establishing them as promising imaging biomarkers and therapeutic targets for precision neuromodulation intervention.

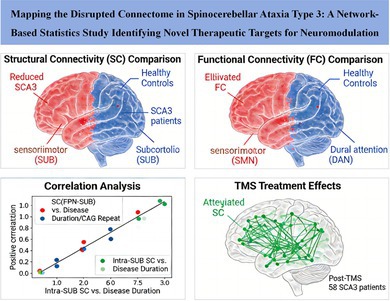

## 1 Introduction

Spinocerebellar ataxia type 3 (SCA3) is the most common autosomal dominant hereditary spinocerebellar ataxia subtype worldwide, accounting for 40%–50% of hereditary ataxia cases [1]. It is caused by an abnormal CAG trinucleotide repeat expansion in the *ATXN3* gene and manifests with progressive motor and non‐motor symptoms [2, 3]. A substantial body of evidence has demonstrated significant cerebellar structural and functional abnormalities in patients with SCA3, solidifying the cerebellum's central role in disease pathology and offering key insights into its primary mechanisms [4, 5, 6, 7]. Nevertheless, the landscape of secondary cerebral network remodeling after the initial cerebellar insult remains poorly understood, and its link to clinical motor symptoms is not fully defined [8]. Given the current lack of curative clinical interventions [9, 10], elucidating the progressive reorganization of the cerebral connectome in SCA3 is of paramount importance for the discovery of novel therapeutic targets.

In recent years, network neuroscience has emerged as a powerful framework for studying neurodegenerative diseases [11, 12]. This approach focuses on brain networks as the primary unit of analysis, aiming to uncover neural mechanisms by quantifying the organizational principles of brain regions and their interconnections [13]. Existing neuroimaging studies have revealed structural impairments within the cerebellar‐basal ganglia‐cortical circuit and abnormal functional connectivity (FC) between the default mode network (DMN) and the frontoparietal network (FPN) in patients with SCA3 [8]. However, region‐specific investigations are insufficient for capturing global, coordinated pathological changes across the brain network [14]. Seed‐based whole‐brain FC analyses have demonstrated significantly reduced connectivity between the DMN and the cerebellar network in patients with SCA3, and this impairment was associated with ataxia severity [15]. Graph theory analyses have been used to quantify alterations in network topology and have revealed partially preserved global network organization in SCA3 [4]. Both global efficiency and nodal efficiency within the cerebellar structural network were reduced in patients with SCA3, with the core hub regions predominantly localized in the anterior and posterior lobes of the cerebellum [5]. Although previous studies have identified cerebro‐cerebellar circuit abnormalities in SCA3, the integrity of inter‐regional connectivity within the cerebrum has yet to be fully characterized [16]. Graph theory analyses, while useful for quantifying network topology, require rigorous multiple‐comparisons correction and cannot precisely identify the core pathological subnetworks [17, 18]. Additionally, how established biomarkers, such as CAG repeat length, relate to subnetwork impairment remains unclear [5, 19]. Despite evidence that transcranial magnetic stimulation (TMS) may modulate abnormal synchronization in the cortical‐basal ganglia‐thalamic circuit and could potentially restore connectivity in SCA3 [20], no study has systematically examined its effects on aberrant subnetworks or its association with clinical improvement. Therefore, this study aims to address these gaps by integrating structural and FC analyses using the network‐based statistic (NBS) approach [21, 22].

We designed a prospective study to assess brain connectivity changes in patients with SCA3 and to test two hypotheses: (1) patients with SCA3 exhibit distinct abnormalities in both structural and functional connectivity, which are associated with CAG repeat length and disease duration; and (2) TMS has the potential to modulate key aberrant connections. Our findings may provide novel insights into the network pathophysiology of SCA3, thereby facilitating the development of disease biomarkers and guiding the formulation of targeted intervention strategies.

## 2 Materials and Methods

**FIGURE 1:**
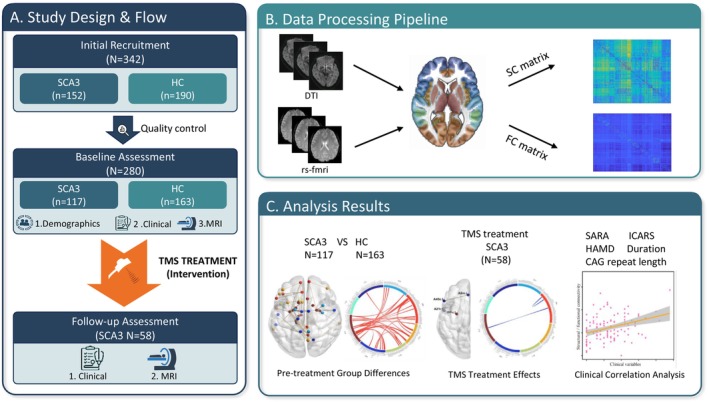
Data generation and analysis workflows. (A) Flowchart of participant selection for SCA3 patients and HCs. (B) Construction of SC and FC networks. DTI preprocessing and SC network construction (denoising, Gibbs ringing artifact removal, eddy current and motion correction, segmentation, gray matter‐white matter boundary creation, inverse transformation of Brainnetome Atlas, SC network construction, SC matrix normalization); rs‐fMRI preprocessing and FC network construction (Slice‐timing correction, inter‐volume motion correction, exclusion of subjects with excessive motion, MNI space normalization, covariate regression, scrubbing, removal, Linear detrending). (C) Network‐based statistics and statistical analysis. DTI, diffusion tensor imaging; FC, functional connectivity; HCs, healthy controls; rs‐fMRI, resting‐state functional magnetic resonance imaging; SC, structural connectivity; SCA3, spinocerebellar ataxia type 3.

The data generation and analysis workflows are shown in Figure 1.

### 2.1 Participants

The study included two study samples: a discovery cohort comprising 117 patients with SCA3 and 163 age‐matched healthy controls (HCs), and a TMS intervention subset comprising 58 patients with SCA3. A detailed description of the stimulation protocol, including localization methods and session structure, is provided in the Supporting Information to ensure full reproducibility. The inclusion criteria for participants with SCA3 were as follows: (1) a diagnosis of SCA3 confirmed by genetic testing and clinical examination; (2) age between 18 and 75 years; (3) the ability to communicate effectively and complete scale assessments independently; and (4) willingness to participate in the study and provide written informed consent. The exclusion criteria for all participants were as follows: (1) presence of other neurodegenerative diseases, such as Parkinson's disease; (2) a history of other serious central nervous system disorders; (3) a history of brain surgery; (4) left‐handedness or having contraindications to MRI; (5) lack of informed consent; and (6) clinically significant depressive symptoms, defined as a Hamilton Depression Rating Scale (HAMD) score ≥ 17, concurrent use of antipsychotic or antidepressant medications, or any pharmacological adjustments, such as initiation, discontinuation, or dose titration, within the preceding 4 weeks. The exclusion criteria for the HCs were as follows: (1) meeting any of the aforementioned exclusion criteria; (2) cognitive impairment, defined as a Montreal Cognitive Assessment (MoCA) score < 26; and (3) major depressive disorder or current use of psychotropic medications.

### 2.2 Clinical, Neuropsychological, and Genetic Assessments

All participants underwent MRI scanning and cognitive assessment using the MoCA. In patients with SCA3, depressive symptoms were additionally assessed using the HAMD, and the severity of ataxia was assessed semi‐quantitatively using the International Cooperative Ataxia Rating Scale (ICARS) and the Scale for the Assessment and Rating of Ataxia (SARA). Genomic DNA was extracted from peripheral blood samples. CAG repeat length in exon 10 of the *ATXN3* gene was determined by polymerase chain reaction (PCR) followed by capillary electrophoresis.

### 2.3 MRI Acquisition

MRI scans were obtained using a Siemens 3T Tim‐Trio MRI scanner (Siemens Healthineers, Forchheim, Germany). Participants were instructed to remain still, keep their eyes closed, stay awake, and avoid thinking about anything in particular. Diffusion tensor imaging data were acquired using the following parameters: TR/TE = 10,000/92 ms, voxel size = 2 × 2 × 2 mm^3^, 65 slices, matrix size = 128 × 128, field of view = 256 × 256 mm^2^, 64 diffusion‐weighted images with *b* = 1000 s/mm^2^, and one non‐diffusion‐weighted image with *b* = 0 s/mm^2^. Resting‐state fMRI data were acquired using the following parameters: TR/TE = 2000/30 ms, 36 slices, flip angle = 90°, voxel size = 3 × 3 × 3 mm^3^, field of view = 192 × 192 mm^2^, and 240 volumes in total. T1‐weighted structural images were acquired using the following parameters: TR/TE = 1900/2.52 ms, TI = 900 ms, flip angle = 9°, voxel size = 1 × 1 × 1 mm^3^, 256 slices, matrix size = 256 × 256, and field of view = 256 × 256 mm^2^.

### 2.4 DTI Preprocessing and SC Network Construction

The diffusion tensor images were processed using MRtrix3 (https://www.mrtrix.org/) and FSL (https://fsl.fmrib.ox.ac.uk/fsl/docs/#/). Briefly, the diffusion tensor images were firstly denoised, followed by removal of Gibbs ringing artifacts. Distortion correction was then performed using the “dwipreproc” command. Subsequently, response functions for different tissue types (white matter, gray matter, and cerebrospinal fluid) were estimated using the “dhollander” algorithm, followed by multi‐tissue constrained spherical deconvolution. The “5ttgen” command was used to segment T1‐weighted images into gray matter, subcortical gray matter, white matter, cerebrospinal fluid, and pathological tissue to generate a five‐tissue‐type image. The gray matter–white matter interface was then used as a seed mask to constrain subsequent streamline analysis. Anatomically constrained probabilistic tractography was performed using the “tckgen” command with default parameters. The resulting streamlines were further filtered using the “tcksift2” command to reduce the over‐representation of certain tracts. The Brainnetome Atlas (Table S1) [23], which consists of 246 regions of interest (ROIs), was inversely transformed from the standard MNI space into each participant's native diffusion space. The “tck2connectome” command was used to construct the structural connectivity (SC) network, resulting in a 246 × 246 SC matrix for each participant. The weights of the SC matrix were defined as streamline counts scaled by the inverse size of the seed and target ROIs, thereby generating a normalized SC matrix.

### 2.5 Resting‐State fMRI Preprocessing and FC Network Construction

Resting‐state fMRI data were preprocessed using the DPABI toolbox [24] (http://rfmri.org/DPABI). Briefly, the first 10 volumes were discarded to allow for participants' adaptation to the scanning environment and stabilization of magnetization. The images were then corrected for slice timing and head motion. Participants with head motion greater than 3 mm in the *x*, *y*, or *z* direction or rotation greater than 3° around any axis were excluded. Next, the resulting images were normalized to standard MNI space using unified segmentation of anatomical images and resampled to a voxel size of 3 × 3 × 3 mm^3^. A multiple regression model was used to remove the effect of covariates of no interest, including 24 motion parameters, white matter signals, and cerebrospinal fluid signals. To further reduce the effects of motion on resting‐state FC, a scrubbing procedure was performed on the preprocessed images [25]. Framewise displacement (FD) was computed for each time point. Time points with excessive motion, defined as an FD greater than 0.5 mm, along with one preceding and two subsequent time points, were regressed out from the time courses. The resulting images were finally linearly detrended and filtered at the range of 0.01–0.1 Hz.

The Brainnetome Atlas was also used to construct the FC network. The mean time course of each ROI was extracted, and Pearson correlation analysis was then used to compute the correlation between each pair of ROIs, resulting in a 246 × 246 FC matrix for each participant. The FC matrix was further Fisher's *z*‐transformed to improve normality.

### 2.6 Statistical Analysis

For the SC network, consistency‐based thresholding was applied to exclude spurious and false‐positive structural connections by retaining only those with a coefficient of variation within the bottom 10th percentile, as suggested by a previous study [26]. Subsequently, the NBS toolbox (http://sites.google.com/site/bctnet/comparison/nbs) was employed to investigate connectivity differences between patients with SCA3 and HCs. Briefly, a primary threshold of *p* < 0.001 (|*t*| > 3.326) was initially applied to the *t*‐statistic computed for each edge to define a set of suprathreshold edges, which were subsequently clustered into connected components (subnetworks). This threshold was selected to identify focal subnetworks with relatively strong effect sizes, as recommended in previous NBS studies [21]. To control the family‐wise error rate (FWER) at the network level, the null distribution of the component size was empirically obtained using a nonparametric permutation approach (5000 permutations). For each permutation, each participant was randomly assigned to one of the two groups, maintaining the same size as the original SCA3 and HCs groups. The same threshold (*t* = 3.326) was then applied to determine the suprathreshold edges, and the maximum component size was computed. A *p*‐value was assigned to each connected component by calculating the proportion of permutations in which the maximum component size was greater than or equal to the observed component size. Subnetworks with FWER corrected *p* < 0.05 were considered significant. Furthermore, to assess the robustness to the choice of primary thresholds, sensitivity analyses were performed by repeating the NBS process using two additional thresholds: *t* = 2.83 (uncorrected *p* = 0.005) and *t* = 3.522 (uncorrected *p* = 0.0005).

For any subnetworks showing altered SC in SCA3 compared to HCs, post hoc analyses were employed to assess the effect of TMS in patients with SCA3 using two‐tailed paired *t*‐tests. Multiple comparisons across altered connections were corrected using the FDR correction at *q* < 0.05.

For the FC network, between‐group differences in FC between patients with SCA3 and HCs were investigated using NBS with permutation‐based FWER correction, following the same procedures described above. For altered functional connections identified by NBS, the effects of TMS were further assessed using two‐tailed paired *t*‐tests, with FDR correction applied for multiple comparisons.

To determine whether the associations between connectivity alterations and clinical variables were independent of demographic factors, we performed partial correlation analyses. Specifically, for the altered SC/FC in patients with SCA3, we investigated their relationships with disease duration and CAG repeat length, adjusting for age, sex, and education as covariates. Furthermore, for the connections significantly affected by TMS, we computed partial correlations between the changes in connectivity strength (pre‐treatment minus post‐treatment) and changes in clinical scores (pre‐treatment minus post‐treatment), including SARA, ICARS, HAMD, and MoCA, using the same covariates to control for potential confounders. Multiple comparisons were corrected using the FDR method (*q* < 0.05).

## 3 Results

Unless otherwise specified, all reported results were corrected for multiple comparisons using the FDR method.

### 3.1 Demographic and Clinical Characteristics of Participants

From an initial pool of 152 screened patients, 117 patients with SCA3 (mean ± standard deviation (SD) age, 43.1 ± 11.6 years; 61 females [52.1%]) were ultimately included. Thirty‐five patients were excluded due to left‐handedness (*n* = 4), incomplete clinical data (*n* = 12), or inadequate image quality (*n* = 19). Additionally, 163 HCs (mean ± SD age, 46.1 ± 13.8 years; 89 females [54.6%]) were recruited. The sociodemographic and clinical profiles of all participants are summarized in Table 1.

**TABLE 1:** Sociodemographic and clinical characteristics of the sample.

Characteristic	Discovery dataset			TMS intervention subset		
	SCA3	HCs	*p*	(SCA3 = 58)		*p*
	(*n* = 117)	(*n* = 163)		Before	After	
Gender (male/female)	56/61	74/89	0.68^a^	—	—	—
Age (year)	43.1 ± 11.6	46.1 ± 13.8	0.06^b^	—	—	—
Education (years)	11.6 ± 3.6	12.0 ± 4.0	0.43^b^	—	—	—
Disease duration (years)	6.5 ± 5.3	—	—	—	—	—
CAG‐repeat expansion	68.9 ± 7.9	—	—	—	—	—
Dystonia	16/117	—	—	—	—	—
Parkinsonism	22/117	—	—	—	—	—
ICARS	30.7 ± 20.0	—	—	39.6 ± 17.8	35.0 ± 18.6	0.00^d^
SARA	10.8 ± 7.6	—	—	14.5 ± 7.8	12.7 ± 7.4	0.00^d^
MoCA score	23.9 ± 5.4	28.7 ± 1.1	0.00^c^	—	—	—
HAMD score	9.2 ± 9.7	—	—	—	—	—

Abbreviations: HAMD, Hamilton depression rating scale; ICARS, International Cooperative Ataxia Rating Scale; MoCA, Montreal cognitive assessment; SARA, Scale for the Assessment and Rating of Ataxia; TMS, Transcranial Magnetic Stimulation.

^a^ Two‐sample chi‐square test.

^b^ Two‐tailed, two‐sample *t*‐test.

^c^ Mann–Whitney *U* test.

^d^ Paired‐samples *t*‐test.

### 3.2 Abnormalities of SC in SCA3

**FIGURE 2:**
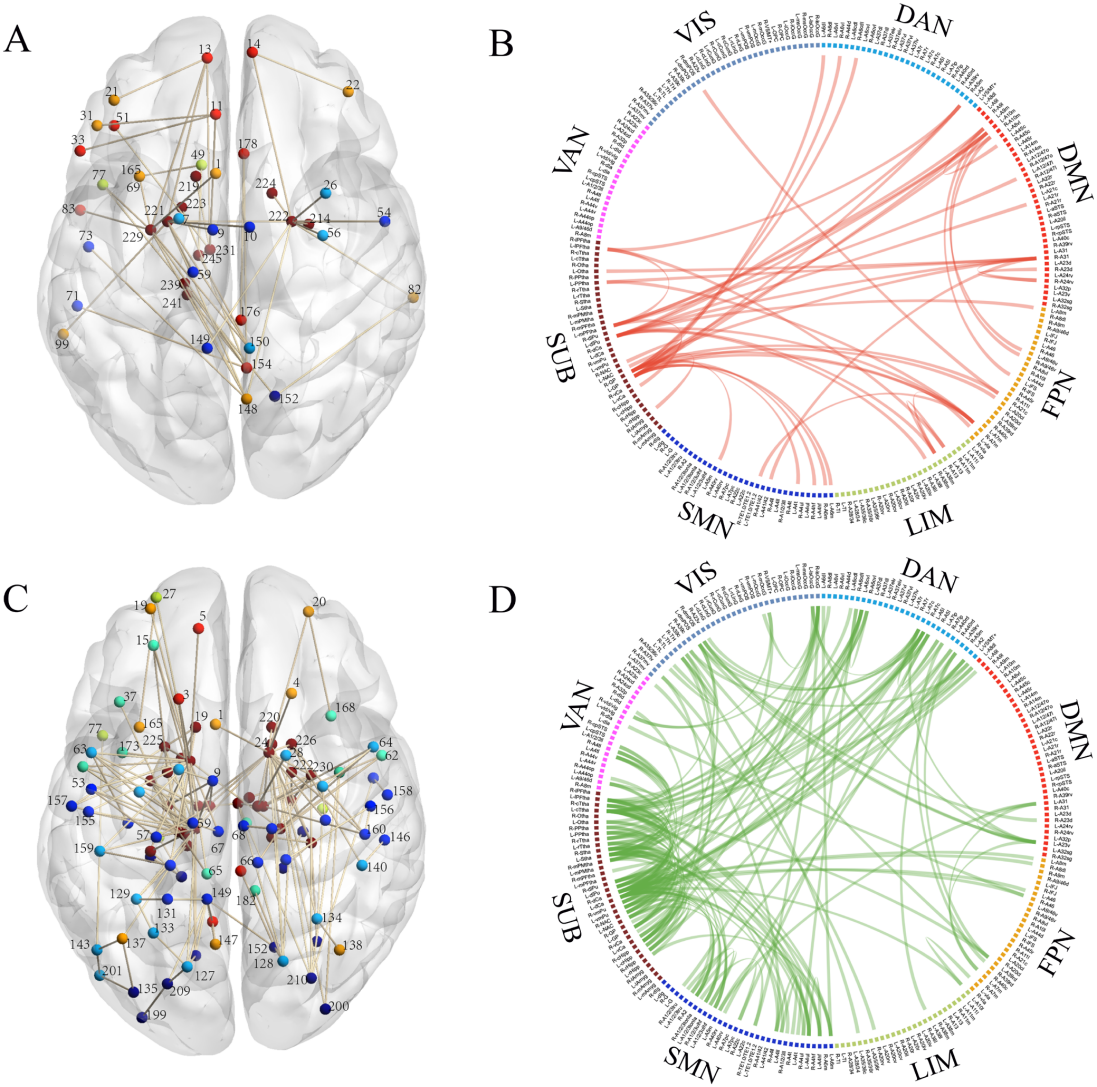
SC abnormalities in participants with SCA3 compared to HCs. (A, C) Brain region level: Visualization maps of NBS subnetworks with significantly increased (A) and decreased (C) SC. Node colors represent specific network modules. (B, D) Network level: Visualization maps of NBS subnetworks with significantly increased (B) and decreased (D) SC, displayed as circular graphs. Edge and node colors represent network hierarchies. For a complete list of regional node abbreviations and their corresponding full anatomical names, please refer to Table S1. HCs, healthy controls; NBS, Network‐based statistics; SC, structural connectivity; SCA3, spinocerebellar ataxia type 3.

Compared to HCs, we identified one subnetwork with enhanced SC and another with reduced SC in SCA3 patients. Specifically, the enhanced SC subnetwork comprised 43 nodes and 48 connections with significantly increased strength (FWER corrected, *p* < 0.001). Regionally, the connections within this subnetwork were observed in frontal cortical regions and subcortical nuclei. At the network level, they primarily involved the subcortical network (SUB), default mode network (DMN), and frontoparietal network (FPN). Notably, the connections with the highest t‐values, specifically between the right dorsal posterior cingulate cortex (A31) and the left dorsolateral putamen (dlPu), and between the left medial area 10 (A10m) and the left medial prefrontal thalamus (mPFtha), represented connectivity between core hubs of the DMN and SUB (Figure 2A,B; Table S2). The reduced SC subnetwork consisted of 104 nodes and 171 connections demonstrating significantly reduced strength (FWER corrected, *p* < 0.001). Regionally, this subnetwork primarily involved subcortical nuclei. At the network level, it was mainly concentrated in the SUB, sensorimotor network (SMN), and dorsal attention network (DAN) (Figure 2C,D; Table S3).

To ensure the robustness of these structural findings, a sensitivity analysis was performed using alternative primary thresholds (*t* = 2.83, *p* = 0.005 and *t* = 3.522, *p* = 0.0005). The analysis consistently identified both significantly increased and decreased structural subnetworks across all thresholds. While the components fragmented into more specific core clusters at the most stringent threshold (*t* = 3.522), the overall pattern and direction of structural reorganization remained highly consistent (Figures [Link], [Link], [Link]).

### 3.3 Abnormalities of FC in SCA3

**FIGURE 3:**
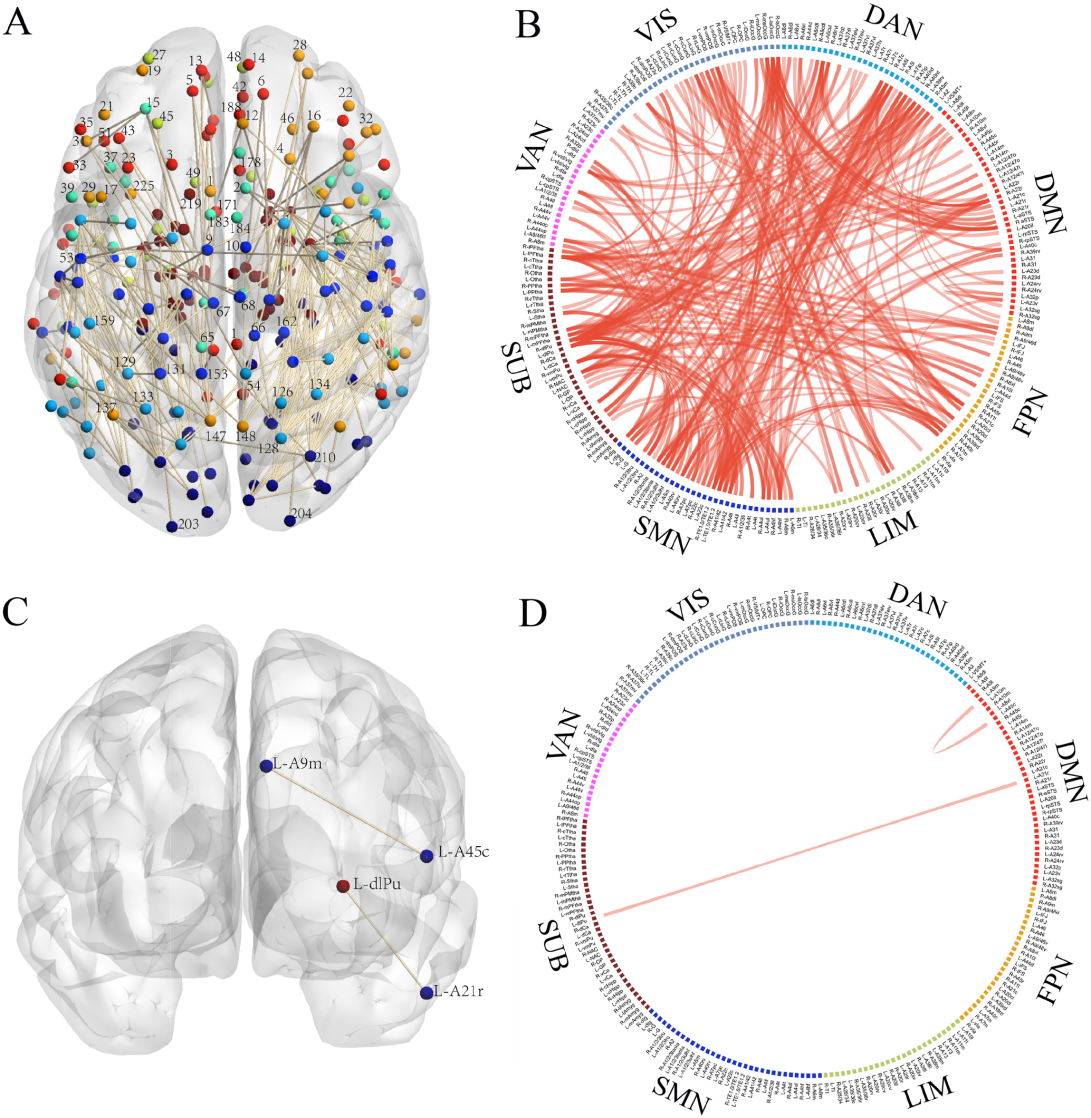
Abnormalities of FC in SCA3; Changes in SC after treatment for SCA3. (A) Brain region level: Visualization map of NBS subnetworks with significantly increased FC, where the color of nodes is determined by the network. (B) Network level: Visualization map of NBS subnetworks with significantly increased FC, where the color of nodes in the circular graph is determined by the network. (C) Brain region level: Visualization of NBS subnetworks where abnormally increased SC at baseline was significantly decreased after treatment, with node colors representing network modules. (D) Network level: Visualization of NBS subnetworks where abnormally increased SC at baseline was significantly decreased after treatment, where node colors in circular graphs represent network hierarchies. For a complete list of regional node abbreviations and their corresponding full anatomical names, please refer to Table S1. FC, functional connectivity; L‐A21r, left rostral middle temporal gyrus; L‐A45c, left caudal inferior frontal gyrus (IFG); L‐A9m, left medial superior frontal gyrus; L‐dlPu, left dorsolateral putamen; NBS, Network‐based statistics.

An abnormal FC subnetwork was identified in SCA3 patients (Figure 3A,B; Table S4), consisting of 162 nodes and 304 connections with increased strength (*p* < 0.001). Regionally, this subnetwork primarily involved the motor cortex and subcortical nuclei. At the network level, it was mainly concentrated in the DAN, SMN, and SUB. The connection exhibiting the highest *t*‐value was directly between the motor cortex and subcortical nuclei, specifically between the left area 4 (head and face region, A4hf) and the left dlPu. Compared to HCs, SCA3 patients did not exhibit any significant reductions in FC. However, a supplementary analysis incorporating global signal regression (GSR) into the preprocessing pipeline revealed a bidirectional pattern, unmasking both localized FC decreases and concurrent increases in SCA3 patients compared to HCs (Figure S4).

### 3.4 Effects of TMS Intervention on SC and FC

Following TMS intervention, patients with SCA3 exhibited significant clinical improvements, characterized by substantial reductions in both SARA (pre‐treatment: 14.5 ± 7.8; post‐treatment: 12.7 ± 7.4, *p* < 0.001) and ICARS scores (pre‐treatment: 39.6 ± 17.8; post‐treatment: 35.0 ± 18.6, *p* < 0.001). Concurrently, two SC connections exhibited significant alterations in SCA3 patients (Figure 3C,D). Specifically, the connection between the left medial superior frontal gyrus (A9m) and the left caudal inferior frontal gyrus (A45c) showed a decrease in strength (pre‐treatment mean = 0.82, post‐treatment mean = 0.63; *t* = −3.27). Similarly, the connection between the left rostral middle temporal gyrus (A21r) and the left dlPu also exhibited a significant decrease (pre‐treatment mean = 0.61, post‐treatment mean = 0.50; *t* = −3.24). However, correlation analyses revealed that the magnitude of these clinical improvements showed no significant associations with the SC changes within these two identified pathways. Notably, no significant alterations in FC were observed following the TMS intervention.

### 3.5 Correlations Between SC/FC Abnormalities and Clinical Variables in SCA3

**FIGURE 4:**
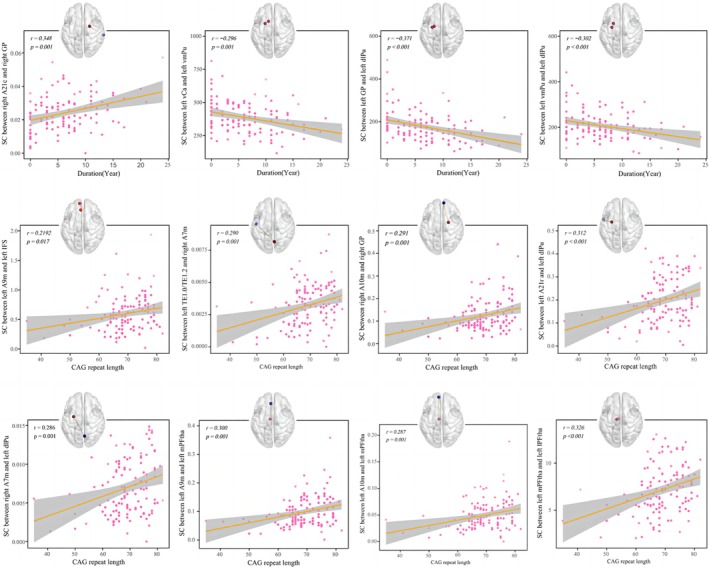
Correlation between structural connectivity and SCA3 clinical features. Disease duration was positively correlated with the structural connectivity between the right A21c and right GP (*r* = 0.348, *p* = 0.001), and negatively correlated with the structural connectivity strength between the left vCa and left vmPu (*r* = −0.296, *p* = 0.001), left GP and left dlPu (*r* = −0.371, *p* < 0.001), and left vmPu and left dlPu (*r* = −0.302, *p* < 0.001). In addition, CAG repeat length was positively correlated with the structural connectivity strength between the left A9m and left IFS (*r* = 0.219, *p* = 0.017), left TE1.0/TE1.2 and right A7m (*r* = 0.290, *p* = 0.001), right A10m and right GP (*r* = 0.291, *p* = 0.001), left A21r and left dlPu (*r* = 0.312, *p* < 0.001), right A7m and left dlPu (*r* = 0.286, *p* = 0.001), left A9m and left mPFtha (*r* = 0.300, *p* = 0.001), left A10m and left mPFtha (*r* = 0.287, *p* = 0.001), and left mPFtha and left lPFtha (*r* = 0.326, *p* < 0.001). A10m, medial area 10; A21c, caudal middle temporal gyrus; A21r, rostral middle temporal gyrus; A7m, medial area 7 (PEp); A9m, medial superior frontal gyrus; dlPu, dorsolateral putamen; GP, globus pallidus; IFS, inferior frontal sulcus; lPFtha, lateral prefrontal thalamus; mPFtha, medial prefrontal thalamus; vCa, ventral caudate; vmPu, ventral medial putamen.

In patients with SCA3, disease duration was negatively correlated with three structural connections that exhibited abnormal reductions (Figure 4). Specifically, the connections between the left ventral caudate nucleus (vCa) and the left ventral medial putamen (vmPu) (*r* = −0.296, *p* = 0.001), between the left globus pallidus (GP) and left dlPu (*r* = −0.371, *p* = 0.0001), and between the left vmPu and the left dlPu (*r* = −0.302, *p* = 0.0003). Conversely, the abnormally enhanced SC between the right caudal middle temporal gyrus (A21c) and the right GP showed a significant positive correlation with disease duration (*r* = 0.348, *p* = 0.001; Figure 4).

CAG repeat length was positively correlated with eight abnormally increased structural connections (Figure 4), including the connection between the left medial prefrontal thalamus (mPFtha) and the left lateral prefrontal thalamus (lPFtha) (*r* = 0.326, *p* = 0.0008), and the connection between the left A21r and the left dlPu (*r* = 0.312, *p* = 0.0004). However, no significant correlations were observed between abnormal FC and disease duration, CAG repeat length, or clinical scale scores.

## 4 Discussion

In this study, we comprehensively investigated alterations in SC and FC in patients with SCA3. We identified distinct network connectivity patterns that differentiate SCA3 patients from HCs. Specifically, SCA3 was characterized by a dual pattern of significantly reduced SC accompanied by enhanced FC within the SUB, SMN, and DAN, as well as increased SC among the SUB, DMN, and FPN. Notably, disease duration and CAG repeat length were specifically associated with SC alterations in the basal ganglia and thalamocortical circuits, whereas FC showed no such correlations. Furthermore, two SC pathways exhibiting abnormal hyperconnectivity at baseline showed significantly reduced connectivity after TMS intervention. Collectively, our findings provide multidimensional insights into the neuropathological mechanisms of brain network disruption in SCA3 and underscore the potential of TMS as a therapeutic strategy.

### 4.1 Bidirectional Structural Reorganization and Functional Hyperconnectivity in SCA3

This study revealed significant alterations in SC patterns between SCA3 patients and HCs. The increased SC observed in SCA3 was concentrated in fronto‐subcortical networks, primarily involving the DMN, SUB, and FPN. These findings are consistent with previous multimodal neuroimaging studies in SCA3 [4]. From a broader connectomic perspective, the disconnection–compensation pattern, characterized by structural integrity loss accompanied by compensatory connectivity enhancement, reflects a generalized network adaptation mechanism observed across neurodegenerative diseases [22]. In various proteinopathies and progressive neurodegenerative disorders, the brain may recruit alternative neural pathways to maintain functional integration during the early stages of pathology [27]. In this dynamic interplay, structural hyperconnectivity does not imply preserved network health; rather, it indicates the recruitment of alternative pathways, reduced network segregation, or compensatory over‐engagement to bypass early primary structural damage [28]. Importantly, the spatial expression of this compensatory mechanism in SCA3 aligns with the disease's specific pathological trajectory. Gray matter atrophy in SCA3 progressively extends from the cerebellar vermis to the frontal cortex and subcortical nuclei, driving cross‐network reorganization [29, 30]. The prominent involvement of the DMN–hypothalamus circuit in our study is particularly noteworthy [31, 32]. This circuit may provide a potential neuroanatomical substrate for non‐motor symptoms frequently observed in SCA3, including sleep disturbances, autonomic dysfunction, and neuroendocrine abnormalities [33, 34].

Concurrently, the decreased SC strength within the SUB, SMN, and DAN may indicate ongoing neurodegenerative damage in SCA3 [35]. The most pronounced impairment involved intrinsic basal ganglia connections, including those between the vCa and vmPu and between the GP and dlPu. This pattern is consistent with evidence that SCA3 preferentially disrupts cerebellar–neostriatal–prefrontal circuitry [36]. Moreover, microstructural damage within cerebellothalamocortical white matter and reduced integrity of basal ganglia pathways have been shown to correlate with motor disability severity [30]. Our findings extend these observations by showing that intrinsic basal ganglia disconnection is accompanied by selective large‐scale network reorganization. This highlights its central role in the pathogenesis of motor dysfunction and refines current models of network‐level pathology in SCA3.

In contrast to the bidirectional SC alterations, FC changes in our main analysis were characterized by a selective increase, particularly within the DAN, SMN, and SUB. This unidirectional hyperconnectivity differs from the mixed connectivity profiles reported in disorders such as Parkinson's disease [37, 38]. Given that our cohort comprised predominantly early‐ to mid‐stage patients, this FC hyperconnectivity may represent a transient adaptive response that helps maintain functional output despite structural compromise. However, this predominantly positive functional profile also necessitates methodological contextualization. Our primary analysis omitted GSR to preserve biologically meaningful neural activity linked to disease burden; however, this approach may shift the correlation distribution positively, emphasizing compensatory hyperconnectivity [39, 40]. Conversely, our supplementary analysis utilizing GSR revealed a bidirectional pattern of targeted FC decreases alongside increases. Rather than being contradictory, these pipelines provide complementary connectomic insights: non‐GSR results capture large‐scale, dominant functional compensation, whereas GSR may increase sensitivity to localized FC reductions [41, 42].

### 4.2 SC as a Potential Imaging Biomarker of TMS‐Induced Network Plasticity in SCA3

Our study provides preliminary evidence that TMS‐associated network modulation in SCA3 involves two key SC pathways within the cortico‐striatal‐cerebellar circuit: the left A9m‐A45c and the left A21r‐dlPu pathways. Consistent with nodal vulnerability theory, the pathological hubs in our study are heavily concentrated within subcortical and sensorimotor networks, anchored by the dlPu, vmPu, and PFtha. Identifying these hubs provides a mechanistic rationale for the observed TMS effects. The modulation of the left A21r‐dlPu pathway is particularly noteworthy: the A21r is a key cortical node for attentional and executive control, whereas the dlPu is the core subcortical sensorimotor hub. This suggests that TMS may facilitate clinical improvement by rebuilding top‐down cognitive and attentional control over motor execution. This finding provides indirect support for the critical involvement of the cortico‐striatal‐cerebellar circuit in SCA3 [15], and also supports the notion that SC at the network level can shape the propagation of TMS‐induced effects [43]. TMS‐induced neuromodulation is highly dependent on the intrinsic functional and structural properties of the targeted neural circuitry [44]. The observed attenuation of hyperconnectivity may reflect a partial normalization of maladaptive network architecture. However, whether this network‐level normalization translates into sustained clinical benefit requires further validation [45]. This work extends previous findings by identifying the specific SC pathways that may serve as candidate substrates for TMS‐induced neuromodulation, thereby addressing a critical gap in understanding the structural basis of TMS therapy for SCA3.

The biological mechanisms through which TMS may attenuate abnormally increased SC remain speculative but may involve activity‐dependent plasticity at multiple levels. First, at the synaptic and circuit levels, repetitive TMS can induce long‐term potentiation or long‐term depression‐like plasticity depending on stimulation parameters [46]. Across repeated sessions, these electrophysiological effects may engage homeostatic plasticity and synaptic remodeling processes, thereby shifting over‐engaged network pathways toward a more physiological state [47, 48]. Second, at the microstructural level, cumulative TMS‐induced neural activity may contribute to activity‐dependent white matter remodeling. Given the widespread impairment of white matter integrity in SCA3 [49], activity‐dependent axonal signaling may influence oligodendrocyte precursor cells, myelination, axonal organization, or extracellular water dynamics [50]. Such changes may be indirectly captured by diffusion‐derived measures of white matter microstructure or tractography‐based connectivity [51]. Nevertheless, because diffusion‐derived SC provides an indirect measure of macroscopic white matter organization rather than direct evidence of synaptic or axonal remodeling [52], these mechanistic interpretations should be considered provisional and require validation in longitudinal, sham‐controlled, and multimodal studies.

Beyond SCA3, this network‐based framework may have broader translational implications for other neurodegenerative diseases. Aberrant connectome topology is increasingly recognized as a shared feature of motor and cognitive decline. In Alzheimer's disease, for example, identifying disease‐related network alterations helps inform therapeutic targeting, biomarker interpretation [53], and intervention strategies [54]. By demonstrating how TMS interacts with specific structural pathways, our findings provide conceptual support for utilizing connectivity‐guided approaches to explore and refine targeted neuromodulation across a wider spectrum of neurological disorders.

### 4.3 Clinical Correlation Analysis

The strength of intrinsic basal ganglia connectivity correlated negatively with disease duration, suggesting that degeneration within the SUB circuitry is a progressive process, which may underlie the gradual deterioration of motor coordination in SCA3 [55]. In contrast, disease duration correlated positively with enhanced connectivity between the right A21c and the right GP. This further supports the dual‐mechanism concept that the corticostriatal compensatory pathways are upregulated to maintain motor control as intrinsic basal ganglia connections diminish [56, 57].

CAG repeat length was positively correlated with the aberrant hyperconnectivity of eight SC pathways localized to the basal ganglia and thalamocortical circuits, thereby providing direct evidence for the association between genetic etiology and brain network remodeling [58]. Notably, previous studies have shown that CAG repeat length is positively correlated with perivascular space burden in the basal ganglia, which reflects the accumulation of the pathogenic ataxin‐3 protein [59]. Our findings further extend this conclusion, confirming that the expanded CAG repeat sequence not only induces damage to subcortical structures but also drives compensatory hyperconnectivity in thalamocortical and corticostriatal circuits [4].

Furthermore, although TMS induced significant structural connectivity changes, these alterations did not correlate with SARA or ICARS scores. This dissociation likely stems from two factors. First, a 4‐week intervention may be sufficient for microstructural remodeling but too brief to yield measurable motor improvements in chronic progressive ataxia, reflecting the temporal lag between neural plasticity and functional recovery [60]. Second, macroscopic observer‐based scales like SARA and ICARS may lack the sensitivity to detect subtle, early‐stage improvements [61]. Thus, the observed structural alterations likely represent early imaging indicators of neuroplasticity that precede overt clinical amelioration. Future studies incorporating longer interventions, extended follow‐ups, and objective kinematic assessments (e.g., wearable sensors) are needed to better capture this brain‐behavior relationship [62].

Several limitations of this study should be noted. First, our baseline analyses are cross‐sectional and the TMS follow‐up is relatively short, limiting causal interpretations of brain‐behavior relationships. Although the identified network metrics show promise as biomarkers for SCA3 progression and treatment response, their clinical applicability requires further validation. Future prospective studies must incorporate independent cohorts, extended follow‐ups, and multimodal imaging to fully establish the robustness and generalizability of these connectivity‐based biomarkers. Second, the absence of a randomized, sham‐controlled design limits the observed SC pathway changes to preliminary associations rather than definitive causal effects. Potential confounders, including placebo responses and MRI test–retest variability, may have influenced these longitudinal findings. Future double‐blind, sham‐controlled trials are therefore essential to validate these causal links. Third, the use of dual stimulation targets and hybrid protocols precludes the isolation of the specific contributions of each component to the observed connectivity changes. These alterations may reflect target‐specific effects or, more likely, a synergistic interaction. Future multi‐arm trials are essential to disentangle these mechanisms and optimize parameters for each target. Finally, the lack of animal‐model validation, particularly using optogenetic manipulation, limits our ability to confirm the mechanisms underlying the identified circuits. Future preclinical studies using targeted neuromodulation should adopt a bench‐to‐bedside approach to clarify the functional and structural circuitry involved in the disease pathophysiology.

## 5 Conclusions

This study reveals a distinct dual pattern of brain network alterations in SCA3, characterized by the coexistence of compensatory hyperconnectivity and progressive degenerative disconnectivity. Importantly, we identified that motor‐related structural connectivity is not only modulable by TMS intervention but also significantly correlated with disease severity (CAG repeat length and disease duration). These findings establish SC as a promising imaging biomarker for monitoring disease progression and therapeutic efficacy and lay a foundation for the development of precision neuromodulation strategies against this disease.

## Author Contributions

C.L., J.W., Z.L., and L.O. designed the study, analyzed the data, and wrote the main manuscript. C.Z. and X.W. analyzed the data and collected the data. L.S. and P.O. collected the data. X.C. and H.C. conducted the scale evaluation. C.X., B.W., and W.C. revised the manuscript. All authors contributed to the article and approved the submitted version.

## Funding

This research was supported by grants from the Young Middle‐aged Senior Medical Talents studio of Chongqing (524Z28921), Chongqing City Key Medical Research Program of Science‐Health Collaboration (2025GGXM005), Senior Medical Talents Program of Chongqing for Young and Middle‐aged (514Z395), Excellent Young Talent Fund of the First Affiliated Hospital of the Army Medical University (2024YQBJ‐2), 2024 Clinical Research Incubation Project of the First Affiliated Hospital of Army Medical University (2024IITZDB10), and Natural Science Foundation of China (82071910, 82572201) provided funding for this study.

## Ethics Statement

The study received approval from the Medical Ethics Committee of the First Affiliated Hospital of Army Medical University (Approval ID: KY2023046 and KY2024203).

## Consent

All participants provided written informed consent to participate in the study.

## Conflicts of Interest

The authors declare no conflicts of interest.

## Supporting information


**Figure S1:** Altered FC in the SCA3 group compared with HCs at different initial thresholds. (A) Initial threshold of *p* = 0.005: The SCA3 group showed increased FC compared with the HCs group. In the circular graph, node colors indicate the network affiliation. (B) Initial threshold of *p* = 0.0005: The SCA3 group showed increased FC compared with the HCs group. In the circular graph, node colors indicate network affiliation. Abbreviations: For a complete list of regional node abbreviations and their corresponding full anatomical names, please refer to Table S1. SCA3 = spinocerebellar ataxia type 3; HCs = healthy controls; FC = functional connectivity.

**Figure S2:** Altered SC in the SCA3 group compared with HCs at an initial threshold of *p* = 0.005. (A) Visualization map of NBS‐identified subnetworks with significantly increased SC. In the circular graph, node colors indicate network affiliation. (B) Visualization map of NBS subnetworks with significantly decreased SC. In the circular graph, node colors indicate network affiliation. Abbreviations: For a complete list of regional node abbreviations and their corresponding full anatomical names, please refer to Table S1. SCA3 = spinocerebellar ataxia type 3; HCs = healthy controls; SC = structural connectivity; NBS = Network‐based statistics.

**Figure S3:** Altered SC in the SCA3 group compared with HCs at an initial threshold of *p* = 0.0005. (A) Visualization map of NBS‐identified subnetworks with significantly increased SC. In the circular graph, node colors indicate network affiliation. Red edges represent subnetwork 1, and green edges represent subnetwork 2. (B) Visualization of NBS‐identified subnetworks with significantly decreased SC. In the circular graph, node colors indicate network affiliation. Abbreviations: For a complete list of regional node abbreviations and their corresponding full anatomical names, please refer to Table S1. SCA3 = spinocerebellar ataxia type 3; HCs = healthy controls; SC = structural connectivity; NBS = Network‐based statistics.

**Figure S4:** Altered functional connectivity in the SCA3 group compared with HCs in the discovery dataset after GSR, at an initial threshold of *p* = 0.001. (A) Visualization of NBS‐identified subnetworks with significantly increased FC. In the circular graph, node colors indicate network affiliation. (B) Visualization of NBS‐identified subnetworks with significantly decreased FC. In the circular graph, node colors indicate network affiliation. Abbreviations: For a complete list of regional node abbreviations and their corresponding full anatomical names, please refer to Table S1. SCA3 = spinocerebellar ataxia type 3; HCs = healthy controls; FC = functional connectivity; GSR = global signal regression; NBS = Network‐based statistics.

**Table S1:** The network definition in Brainnetome atlas.
**Table S2:** Structural Connectivity (SCA3 > HC) showing significant difference between SCA3 and HC in the discovery dataset.
**Table S3:** Structural Connectivity (SCA3 < HC) showing significant difference between SCA3 and HC in the discovery dataset.
**Table S4:** Functional Connectivity (SCA3 > HC) showing significant difference between SCA3 and HC in the discovery dataset.

**Data S1:** Supporting Information.

## Data Availability

The data that support the findings of this study are available from the corresponding author upon reasonable request.
