# Supplementary figures and images for: Mapping the Disrupted Connectome in Spinocerebellar Ataxia Type 3: A Network‐Based Statistics Study Identifying Novel Therapeutic Targets for Neuromodulation
Source: CNS Neurosci Ther. 2026 Jul 6;32(7):e71016. doi: 10.1002/cns.71016 (PMC13334373; doi:10.1002/cns.71016)

A

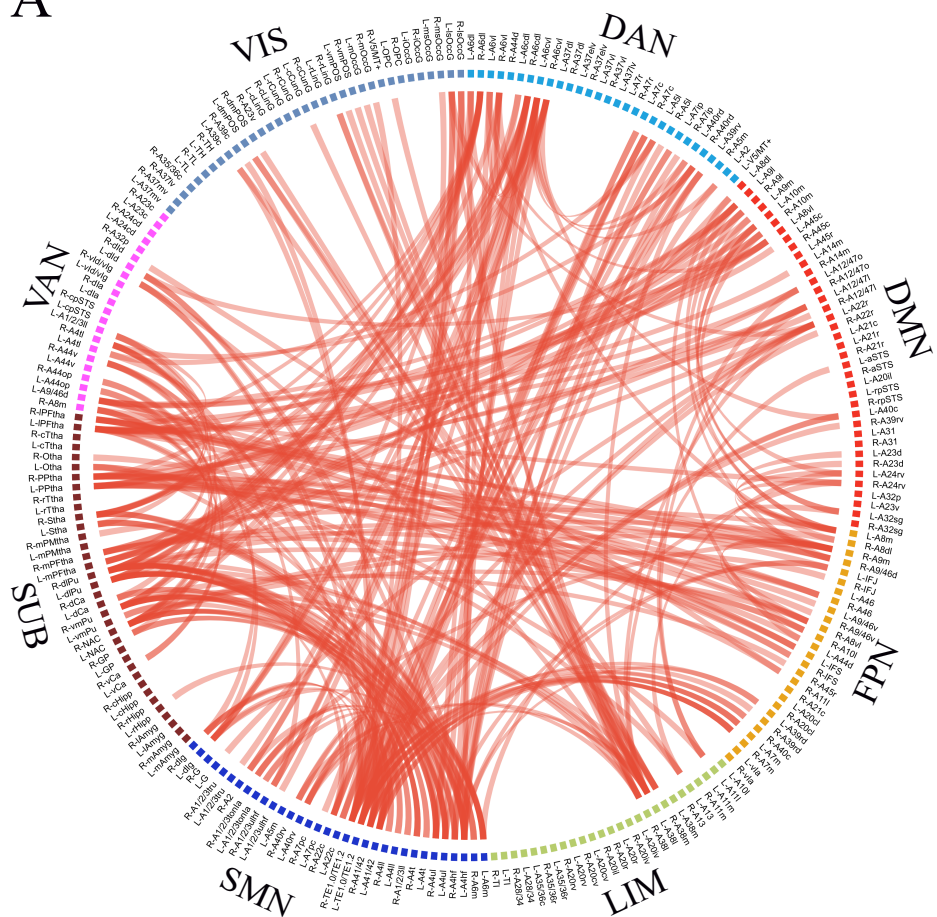

B

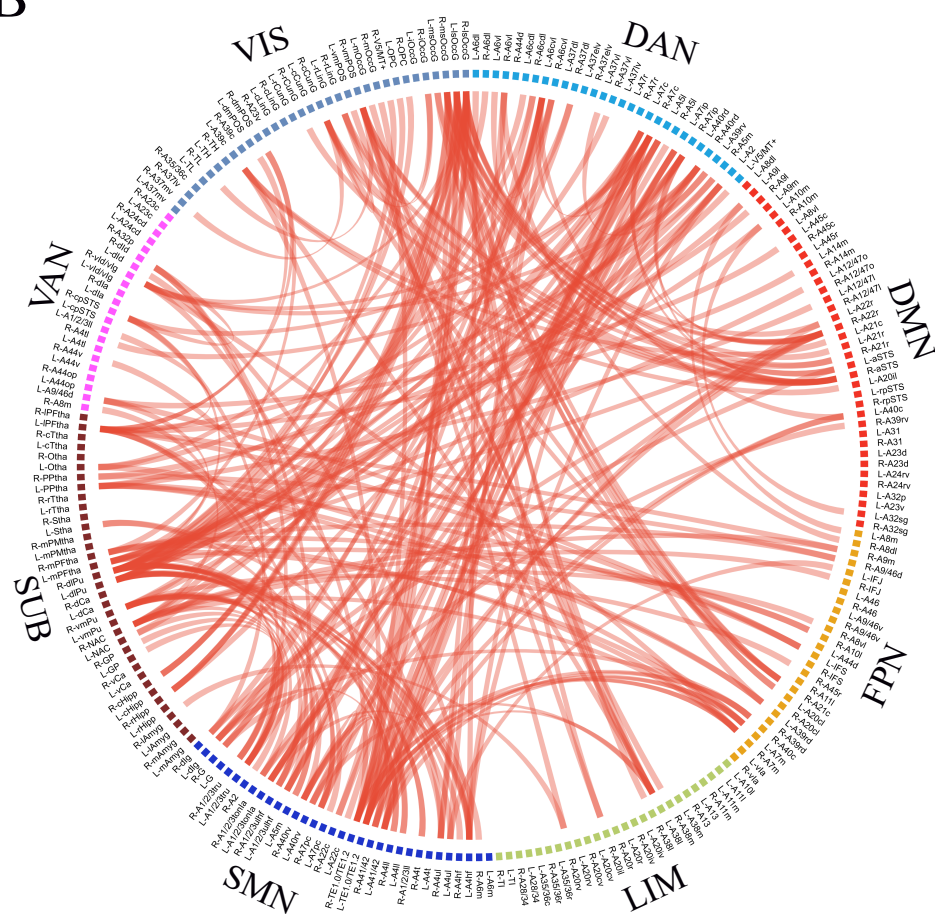

Supplement: Supplementary file 1 — Figure S1: Altered FC in the SCA3 group compared with HCs at different initial thresholds. (A) Initial threshold of p = 0.005: The SCA3 group showed increased FC compared with the HCs group. In the circular graph, node colors indicate the network affiliation. (B) Initial threshold of p = 0.0005: The SCA3 group showed increased FC compared with the HCs group. In the circular graph, node colors indicate network affiliation. Abbreviations: For a complete list of regional node abbreviations and their corresponding full anatomical names, please refer to Table S1. SCA3 = spinocerebellar ataxia type 3; HCs = healthy controls; FC = functional connectivity. [file CNS-32-e71016-s002.pdf]

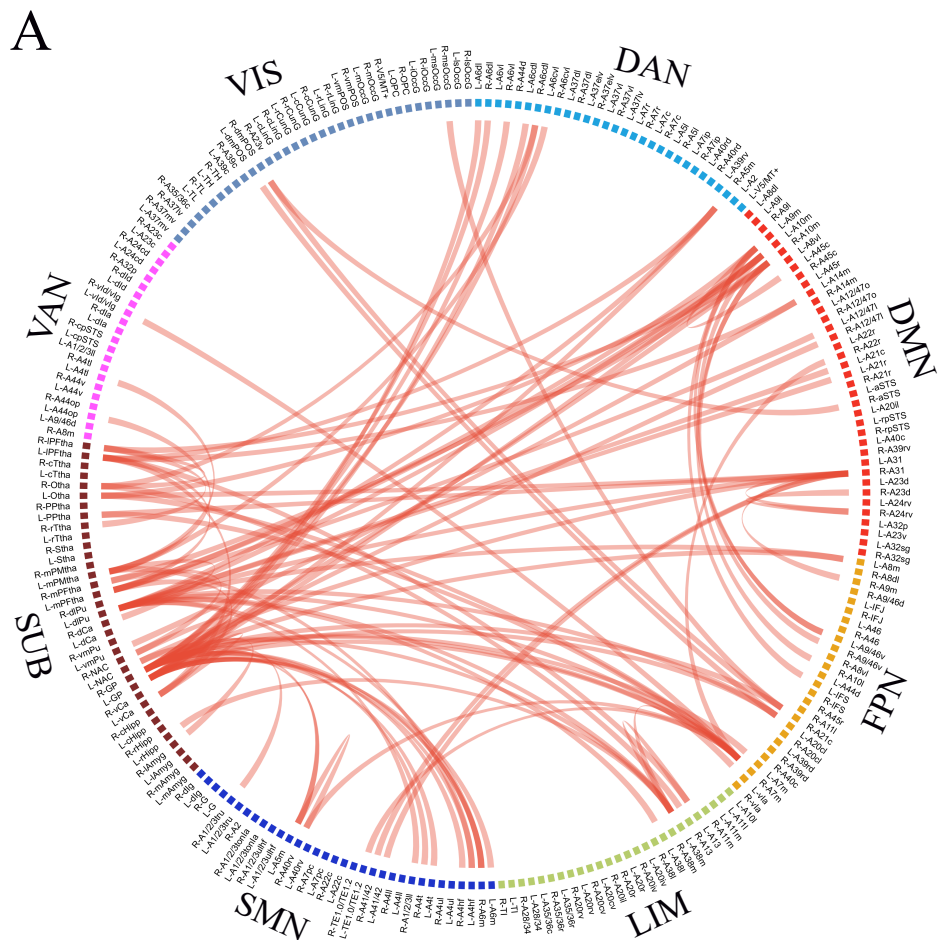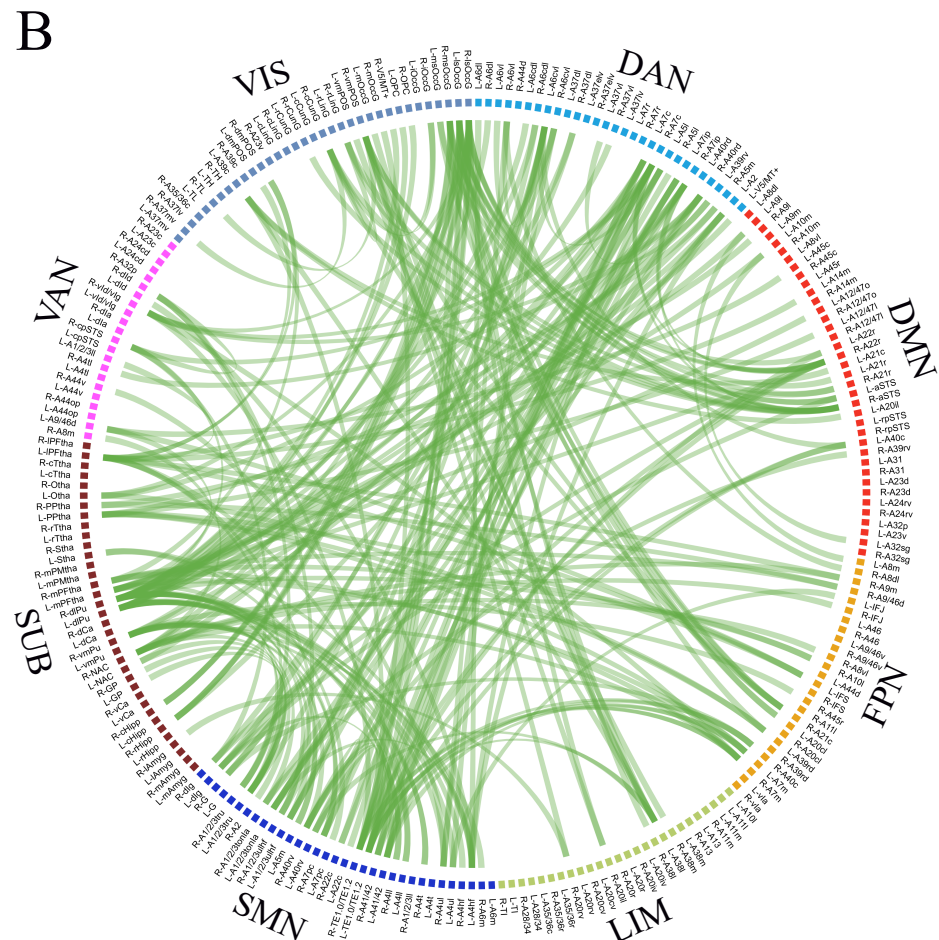

Supplement: Supplementary file 2 — Figure S2: Altered SC in the SCA3 group compared with HCs at an initial threshold of p = 0.005. (A) Visualization map of NBS‐identified subnetworks with significantly increased SC. In the circular graph, node colors indicate network affiliation. (B) Visualization map of NBS subnetworks with significantly decreased SC. In the circular graph, node colors indicate network affiliation. Abbreviations: For a complete list of regional node abbreviations and their corresponding full anatomical names, please refer to Table S1. SCA3 = spinocerebellar ataxia type 3; HCs = healthy controls; SC = structural connectivity; NBS = Network‐based statistics. [file CNS-32-e71016-s001.pdf]

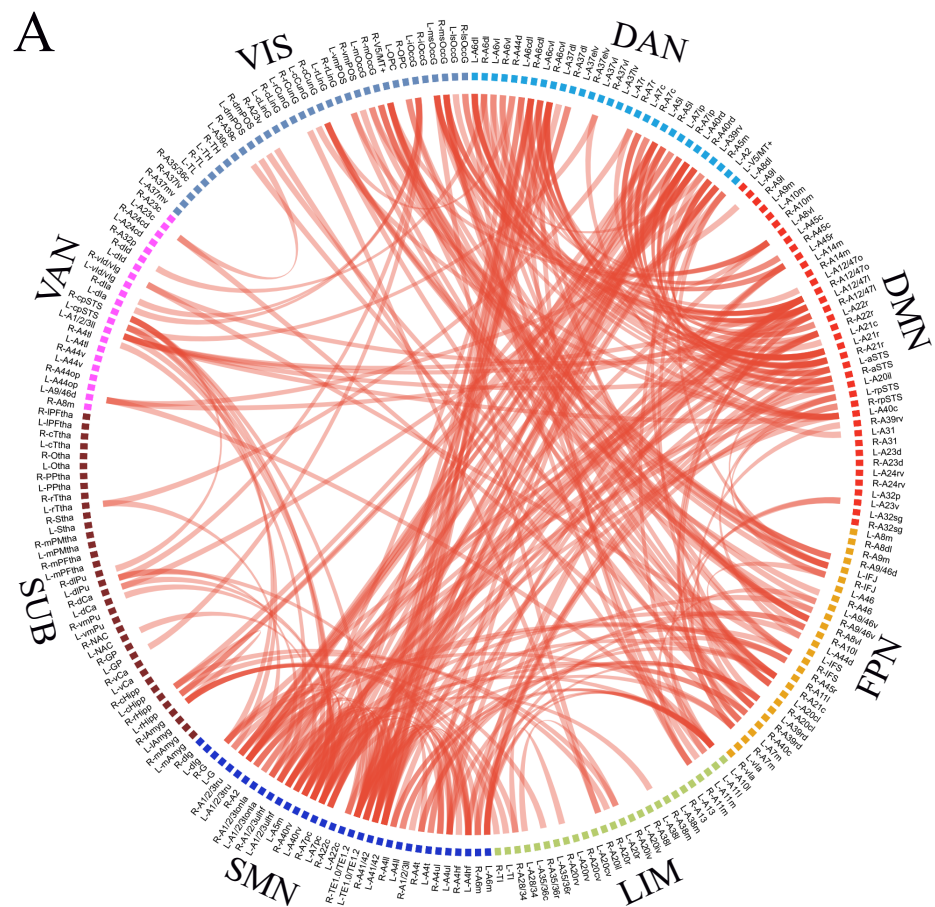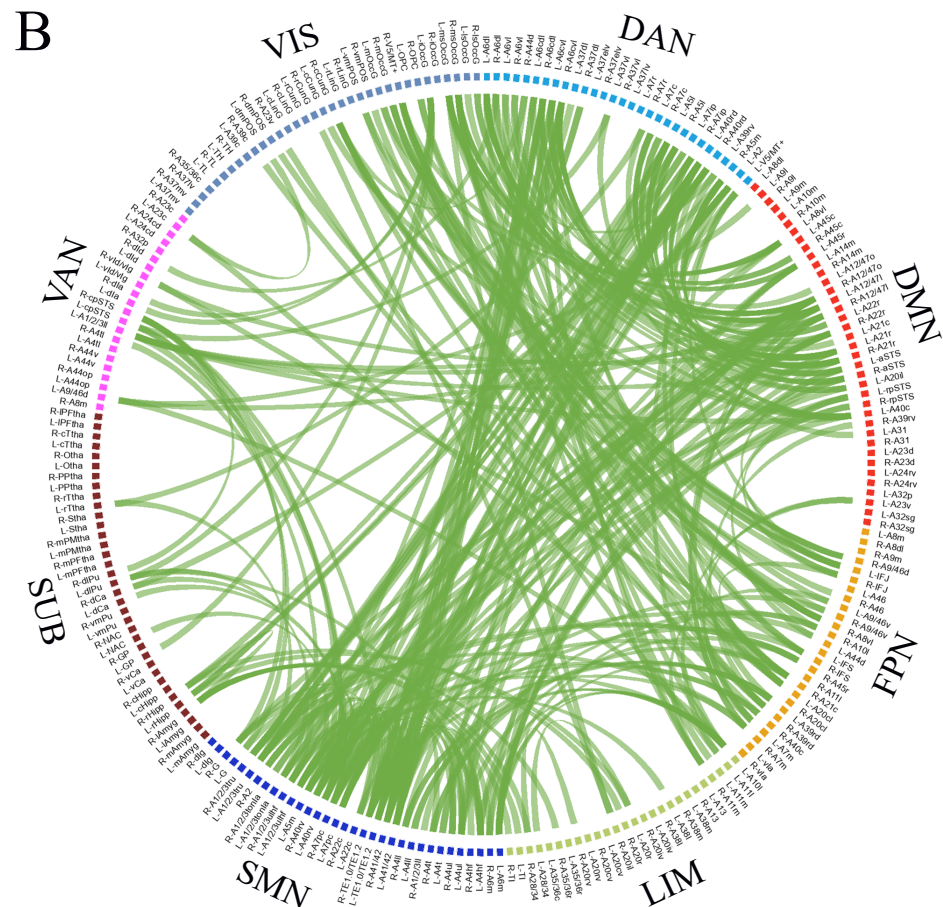

Supplement: Supplementary file 4 — Figure S4: Altered functional connectivity in the SCA3 group compared with HCs in the discovery dataset after GSR, at an initial threshold of p = 0.001. (A) Visualization of NBS‐identified subnetworks with significantly increased FC. In the circular graph, node colors indicate network affiliation. (B) Visualization of NBS‐identified subnetworks with significantly decreased FC. In the circular graph, node colors indicate network affiliation. Abbreviations: For a complete list of regional node abbreviations and their corresponding full anatomical names, please refer to Table S1. SCA3 = spinocerebellar ataxia type 3; HCs = healthy controls; FC = functional connectivity; GSR = global signal regression; NBS = Network‐based statistics. [file CNS-32-e71016-s003.pdf]
