# Supplementary material for: Mapping the Disrupted Connectome in Spinocerebellar Ataxia Type 3: A Network‐Based Statistics Study Identifying Novel Therapeutic Targets for Neuromodulation
Source: CNS Neurosci Ther. 2026 Jul 6;32(7):e71016. doi: 10.1002/cns.71016 (PMC13334373; doi:10.1002/cns.71016)
Supplement: Supplementary file 5 — Table S1: The network definition in Brainnetome atlas. Table S2: Structural Connectivity (SCA3 > HC) showing significant difference between SCA3 and HC in the discovery dataset. Table S3: Structural Connectivity (SCA3 < HC) showing significant difference between SCA3 and HC in the discovery dataset. Table S4: Functional Connectivity (SCA3 > HC) showing significant difference between SCA3 and HC in the discovery dataset. [file CNS-32-e71016-s004.docx]

**Table S1. The network definition in Brainnetome atlas.**

| **ID** | **Description** | **Subregion** | **Network** | **ID** | **Description** | **Subregion** | **Network** |
| --- | --- | --- | --- | --- | --- | --- | --- |
| 1 | A8m | SFG_L_7_1 | FPN | 2 | A8m | SFG_R_7_1 | VAN |
| 3 | A8dl | SFG_L_7_2 | DMN | 4 | A8dl | SFG_R7_2 | FPN |
| 5 | A91 | SFG_L_7_3 | DMN | 6 | A91 | SFG_R_7_3 | DMN |
| 7 | A6dl | SFG_L_7_4 | DAN | 8 | A6dl | SFG_R7_4 | DAN |
| 9 | A6m | SFG_L_7_5 | SMN | 10 | A6m | SFG_R_7_5 | SMN |
| 11 | A9m | SFG_L_7_6 | DMN | 12 | A9m | SFG_R_7_6 | FPN |
| 13 | A10m | SFG_L_7_7 | DMN | 14 | A10m | SFG_R7_7 | DMN |
| 15 | A9/46d | MFG_L7_1 | VAN | 16 | A9/46d | MFG_R_7_1 | FPN |
| 17 | IFJ | MFG_L7_2 | FPN | 18 | IFJ | MFG_R_7_2 | FPN |
| 19 | A46 | MFG_L_7_3 | FPN | 20 | A46 | MFG_R_7_3 | FPN |
| 21 | A9/46v | MFG_L7_4 | FPN | 22 | A9/46v | MFG_R_7_4 | FPN |
| 23 | A8vl | MFG_L_7_5 | DMN | 24 | A8vl | MFG_R_7_5 | FPN |
| 25 | A6vl | MFG_L7_6 | DAN | 26 | A6vl | MFG_R_7_6 | DAN |
| 27 | A101 | MFG_L7_7 | LIM | 28 | A101 | MFG_R_7_7 | FPN |
| 29 | A44d | IFG_L_6_1 | FPN | 30 | A44d | IFG_R_6_1 | DAN |
| 31 | IFS | IFGL_6_2 | FPN | 32 | IFS | IFG_R_6_2 | FPN |
| 33 | A45c | IFG_L_6_3 | DMN | 34 | A45c | IFG_R_6_3 | DMN |
| 35 | A45r | IFG_L_6_4 | DMN | 36 | A45r | IFG_R_6_4 | FPN |
| 37 | A44op | IFGL_6_5 | VAN | 38 | A44op | IFG_R_6_5 | VAN |
| 39 | A44v | IFGL_6_6 | VAN | 40 | A44v | IFG_R_6_6 | VAN |
| 41 | A14m | OrG_L_6_1 | DMN | 42 | A14m | OrG_R_6_1 | DMN |
| 43 | A12/47o | OrG_L_6_2 | DMN | 44 | A12/470 | OrG_R_6_2 | DMN |
| 45 | A111 | OrG_L_63 | LIM | 46 | A11l | OrG_R_6_3 | FPN |
| 47 | Al1m | OrG_L_6_4 | LIM | 48 | Allm | OrG_R_6_4 | LIM |
| 49 | A13 | OrG_L_6_5 | LIM | 50 | A13 | OrG_R_6_5 | LIM |
| 51 | A12/471 | OrG_L_6_6 | DMN | 52 | A12/471 | OrG_R_6_6 | DMN |
| 53 | A4hf | PrGL_6_1 | SMN | 54 | A4hf | PrG_R_6_1 | SMN |
| 55 | A6cdl | PrG_L_6_2 | DAN | 56 | A6odl | PrG_R_6_2 | DAN |
| 57 | A4ul | PrGL_6_3 | SMN | 58 | A4ul | PrG_R_6_3 | SMN |
| 59 | A4t | PrG_L_6_4 | SMN | 60 | A4t | PrG_R_6_4 | SMN |
| 61 | A4tl | PrG_L_6_5 | VAN | 62 | A4tl | PrG_R_6_5 | VAN |
| 63 | A6cvl | PrG_L_6_6 | DAN | 64 | A6cvl | PrG_R_6_6 | DAN |
| 65 | A1/2/3ll | PCL_L2_1 | VAN | 66 | A1/2/3ll | PCL_R_2_1 | SMN |
| 67 | A41l | PCL_L_2_2 | SMN | 68 | A4I | PCL_R_2_2 | SMN |
| 69 | A38m | STGL_6_1 | LIM | 70 | A38m | STG_R_6_1 | LIM |
| 71 | A41/42 | STG_L_6_2 | SMN | 72 | A41/42 | STG_R_6_2 | SMN |
| 73 | TE1.0/TE1.2 | STGL_6_3 | SMN | 74 | TE1.0/TE1.2 | STG_R_6_3 | SMN |
| 75 | A22c | STG_L_6_4 | SMN | 76 | A22c | STG_R_6_4 | SMN |
| 77 | A381 | STG_L_6_5 | LIM | 78 | A381 | STG_R_6_5 | LIM |
| 79 | A22r | STG_L_6_6 | DMN | 80 | A22r | STG_R_6_6 | DMN |
| 81 | A21c | MTG_L_4_1 | DMN | 82 | A21c | MTG_R_4_1 | FPN |
| 83 | A21r | MTG_L_4_2 | DMN | 84 | A21r | MTG_R_4_2 | DMN |
| 85 | A37dl | MTG_L_4_3 | DAN | 86 | A37dl | MTG_R4_3 | DAN |
| 87 | aSTS | MTG_L_4_4 | DMN | 88 | aSTS | MTG_R_4_4 | DMN |
| 89 | A20iv | ITG_L_7_1 | LIM | 90 | A20iv | ITG_R_7_1 | LIM |
| 91 | A37elv | ITG_L7_2 | DAN | 92 | A37elv | ITG_R7_2 | DAN |
| 93 | A20r | ITG_L_7_3 | LIM | 94 | A20r | ITG_R_7_3 | LIM |
| 95 | A20il | ITG_L_7_4 | DMN | 96 | A20il | ITG_R_7_4 | LIM |
| 97 | A37vl | ITG_L_7_5 | DAN | 98 | A37vl | ITGR_7_5 | DAN |
| 99 | A20cl | ITG_L_7_6 | FPN | 100 | A20cl | ITG_R_7_6 | FPN |
| 101 | A20cv | ITG_L_7_7 | LIM | 102 | A20cv | ITGR_7_7 | LIM |
| 103 | A20rv | FuG_L_3_1 | LIM | 104 | A20rv | FuG_R_3_1 | LIM |
| 105 | A37mv | FuG_L_3_2 | VIS | 106 | A37mv | FuG_R_3_2 | VIS |
| 107 | A37lv | FuG_L_3_3 | DAN | 108 | A37lv | FuG_R_3_3 | VIS |
| 109 | A35/36r | PhG L 6 1 | LIM | 110 | A35/36r | PhG R 6 1 | LIM |
| 111 | A35/36c | PhG L 6 2 | LIM | 112 | A35/36c | PhG R 6 2 | VIS |
| 113 | TL | PhG L 6 3 | VIS | 114 | TL | PhG R 6 3 | VIS |
| 115 | A28/34 | PhG L 6 4 | LIM | 116 | A28/34 | PhG R 6 4 | LIM |
| 117 | TI | PhG L 6 5 | LIM | 118 | TI | PhG R 6 5 | LIM |
| 119 | TH | PhG L 6 6 | VIS | 120 | TH | PhG R 6 6 | VIS |
| 121 | rpSTS | pSTS L 2 1 | DMN | 122 | rpSTS | pSTS R 2 1 | DMN |
| 123 | cpSTS | pSTS L 2 2 | VAN | 124 | cpSTS | pSTS R 2 2 | VAN |
| 125 | A7r | SPL_L_5_1 | DAN | 126 | A7r | SPL_R_5_1 | DAN |
| 127 | A7c | SPL_L_5_2 | DAN | 128 | A7c | SPL_R_5_2 | DAN |
| 129 | A51 | SPL_L_5_3 | DAN | 130 | A5l | SPL_R5_3 | DAN |
| 131 | A7pc | SPL_L_5_4 | SMN | 132 | A7pc | SPL_R_5_4 | SMN |
| 133 | A7ip | SPL_L_5_5 | DAN | 134 | A7ip | SPL_R5_5 | DAN |
| 135 | A39c | IPL_L_6_1 | VIS | 136 | A39c | IPL_R_6_1 | VIS |
| 137 | A39rd | IPLL_6_2 | FPN | 138 | A39rd | IPLR62 | FPN |
| 139 | A40rd | IPLL_6_3 | DAN | 140 | A40rd | IPL_R_6_3 | DAN |
| 141 | A40c | IPL_L_6_4 | DMN | 142 | A40c | IPL_R_6_4 | FPN |
| 143 | A39rv | IPL_L_6_5 | DAN | 144 | A39rv | IPL_R_6_5 | DMN |
| 145 | A40rv | IPL_L_6_6 | SMN | 146 | A40rv | IPL_R_6_6 | SMN |
| 147 | A7m | PCun_L4_1 | FPN | 148 | A7m | PCun_R4_1 | FPN |
| 149 | A5m | PCun_L_4_2 | SMN | 150 | A5m | PCun_R_4_2 | DAN |
| 151 | dmPOS | PCun_L_4_3 | VIS | 152 | dmPOS | PCun_R4_3 | VIS |
| 153 | A31 | PCun_L_4_4 | DMN | 154 | A31 | PCun_R4_4 | DMN |
| 155 | A1/2/3ulhf | PoGL_4_1 | SMN | 156 | A1/2/3ulhf | PoG_R4_1 | SMN |
| 157 | A1/2/3tonla | PoGL_4_2 | SMN | 158 | A1/2/3tonla | PoG_R4_2 | SMN |
| 159 | A2 | PoG_L_4_3 | DAN | 160 | A2 | PoG_R_4_3 | SMN |
| 161 | A1/2/3tru | PoG_L_4_4 | SMN | 162 | A1/2/3tru | PoG_R4_4 | SMN |
| 163 | G | INS_L_6_1 | SMN | 164 | G | INS_R_6_1 | SMN |
| 165 | vla | INSL_6_2 | SUB | 166 | vla | INS_R_6_2 | FPN |
| 167 | dla | INS_L_6_3 | VAN | 168 | dIa | INS_R_6_3 | VAN |
| 169 | vld/vIg | INS_L_6_4 | VAN | 170 | vIdvIg | INS_R_6_4 | VAN |
| 171 | dlg | INSL_6_5 | SMN | 172 | dig | INS_R_6_5 | SMN |
| 173 | dld | INSL_6_6 | VAN | 174 | dId | INS_R_6_6 | VAN |
| 175 | A23d | CG_L_7_1 | DMN | 176 | A23d | CG_R_7_1 | DMN |
| 177 | A24rv | CG_L_7_2 | VAN | 178 | A24rv | CG_R_7_2 | DMN |
| 179 | A32p | CG_L_7_3 | DMN | 180 | A32p | CG_R_7_3 | VAN |
| 181 | A23v | CG_L_7_4 | DMN | 182 | A23v | CG_R_7_4 | VIS |
| 183 | A24cd | CG_L_7_5 | VAN | 184 | A24cd | CG_R_7_5 | VAN |
| 185 | A23c | CG_L_7_6 | VAN | 186 | A23c | CG_R_7_6 | VAN |
| 187 | A32sg | CG_L_7_7 | DMN | 188 | A32sg | CG_R_7_7 | DMN |
| 189 | cLinG | MVOcC_L_5_1 | VIS | 190 | cLinG | MVOcC_R_5_1 | VIS |
| 191 | rCunG | MVOcC_L_5_2 | VIS | 192 | rCumG | MVOcC_R5_2 | VIS |
| 193 | cCunG | MVOcC_L_5_3 | VIS | 194 | cCunG | MVOcC_R5_3 | VIS |
| 195 | ILinG | MVOcCL_5_4 | VIS | 196 | rLinG | MVOcC_R5_4 | VIS |
| 197 | vmPOS | MVOcC_L_5_5 | VIS | 198 | vmPOS | MVOcC_R_5_5 | VIS |
| 199 | mOcG | LOcC_L_4_1 | VIS | 200 | mOccG | LOcC_R_4_1 | VIS |
| 201 | V5/MT+ | LOcC_L4_2 | DAN | 202 | V5/MT+ | LOcC_R4_2 | VIS |
| 203 | OPC | LOcC_L_4_3 | VIS | 204 | OPC | LOcC_R_4_3 | VIS |
| 205 | iOccG | LOcC_L_4_4 | VIS | 206 | iOccG | LOcC_R4_4 | VIS |
| 207 | msOccG | LOcC_L_2_1 | VIS | 208 | msOccG | LOcC_R_2_1 | VIS |
| 209 | l₈OccG | LOcC_L2_2 | VIS | 210 | lsOccG | LOcC_R2_2 | VIS |
| 211 | mAmyg | Amyg L 2 1 | SUB | 212 | mAmyg | Amyg R 2 1 | SUB |
| 213 | lAmyg | Amyg L 2 2 | SUB | 214 | lAmyg | Amyg R 2 2 | SUB |
| 215 | THipp | Hipp L 2 1 | SUB | 216 | rHipp | Hipp R 2 1 | SUB |
| 217 | cHipp | Hipp L 2 2 | SUB | 218 | cHipp | Hipp R 2 2 | SUB |
| 219 | vCa | BG_L_6_1 | SUB | 220 | vCa | BG_R_6_1 | SUB |
| 221 | GP | BG_L_6_2 | SUB | 222 | GP | BG_R_6_2 | SUB |
| 223 | NAC | BG_L_63 | SUB | 224 | NAC | BG_R_6_3 | SUB |
| 225 | vmPu | BG_L_6_4 | SUB | 226 | vmPu | BG_R_6_4 | SUB |
| 227 | dCa | BG_L_6_5 | SUB | 228 | dCa | BG_R_6_5 | SUB |
| 229 | dlPu | BG_L_6_6 | SUB | 230 | dlPu | BG_R6_6 | SUB |
| 231 | mPFtha | Tha_L_8_1 | SUB | 232 | mPFtha | Tha_R_8_1 | SUB |
| 233 | mPMtha | Tha_L_8_2 | SUB | 234 | mPMtha | Tha_R_8_2 | SUB |
| 235 | Stha | Tha_L_8_3 | SUB | 236 | Stha | Tha_R_8_3 | SUB |
| 237 | rTtha | Tha_L_8_4 | SUB | 238 | rTtha | Tha_R_8_4 | SUB |
| 239 | PPtha | Tha_L_8_5 | SUB | 240 | PPtha | Tha_R8_5 | SUB |
| 241 | Otha | Tha_L_8_6 | SUB | 242 | Otha | Tha_R_8_6 | SUB |
| 243 | cTtha | Tha_L_8_7 | SUB | 244 | cTtha | Tha_R_8_7 | SUB |
| 245 | PFtha | Tha_L_8_8 | SUB | 246 | PFtha | Tha_R_8_8 | SUB |

VIS=visual network; SMN=somatomotor network; DAN=dorsal attention network; VAN=ventral attention network; LIM=limbic network; FPN=frontoparietal network; DMN=default mode network; SUB=subcortical network.

**Table S2. Structural Connectivity (SCA3＞HC) showing significant difference between SCA3 and HC in the discovery dataset.**

| **ROI label** | **ROI label** | **'Mean-SCA3'** | **'Mean-HC'** | **'T-value'** |
| --- | --- | --- | --- | --- |
| 13 | 21 | 1.21 | 0.83 | 5.4 |
| 14 | 22 | 0.97 | 0.73 | 4.31 |
| 11 | 31 | 0.82 | 0.65 | 3.9 |
| 11 | 33 | 0.64 | 0.51 | 3.67 |
| 49 | 69 | 0.83 | 0.63 | 3.93 |
| 73 | 148 | 0.06 | 0.05 | 3.84 |
| 82 | 152 | 0.32 | 0.24 | 4.4 |
| 71 | 154 | 0.08 | 0.06 | 3.46 |
| 69 | 165 | 2.3 | 1.72 | 4.15 |
| 154 | 178 | 1.32 | 1.01 | 5.05 |
| 176 | 178 | 4.84 | 4.04 | 3.89 |
| 7 | 221 | 1.4 | 1.14 | 3.42 |
| 9 | 221 | 1.16 | 0.94 | 4.27 |
| 10 | 221 | 0.21 | 0.16 | 4.19 |
| 13 | 221 | 0.47 | 0.38 | 3.78 |
| 59 | 221 | 1.27 | 0.97 | 3.91 |
| 99 | 221 | 0.08 | 0.06 | 4.04 |
| 148 | 221 | 0.05 | 0.03 | 4.63 |
| 150 | 221 | 0.05 | 0.04 | 4.47 |
| 14 | 222 | 0.38 | 0.29 | 3.85 |
| 26 | 222 | 1.27 | 0.99 | 3.61 |
| 54 | 222 | 2.37 | 2.01 | 3.43 |
| 56 | 222 | 2.72 | 2.2 | 3.79 |
| 82 | 222 | 0.17 | 0.13 | 4.9 |
| 148 | 222 | 0.25 | 0.19 | 4.22 |
| 149 | 222 | 0.07 | 0.06 | 4.23 |
| 214 | 222 | 3.75 | 2.53 | 5.68 |
| 221 | 222 | 0.57 | 0.45 | 3.63 |
| 154 | 223 | 0.09 | 0.07 | 3.62 |
| 222 | 224 | 12.18 | 9.91 | 3.88 |
| 1 | 229 | 1.41 | 1.18 | 3.69 |
| 13 | 229 | 0.76 | 0.6 | 4.19 |
| 69 | 229 | 0.75 | 0.55 | 4.74 |
| 77 | 229 | 1.34 | 1.06 | 3.76 |
| 83 | 229 | 0.48 | 0.36 | 4.36 |
| 148 | 229 | 0.09 | 0.07 | 4.11 |
| 150 | 229 | 0.1 | 0.08 | 3.83 |
| 154 | 229 | 0.1 | 0.06 | 6.05 |
| 11 | 231 | 0.34 | 0.25 | 5.1 |
| 13 | 231 | 0.25 | 0.17 | 5.77 |
| 51 | 231 | 0.44 | 0.32 | 4.67 |
| 219 | 231 | 4.43 | 3.8 | 3.61 |
| 148 | 239 | 0.14 | 0.11 | 4.21 |
| 154 | 239 | 0.15 | 0.11 | 5 |
| 154 | 241 | 0.13 | 0.09 | 4.67 |
| 49 | 245 | 1.9 | 1.4 | 4.64 |
| 150 | 245 | 0.06 | 0.05 | 3.6 |
| 231 | 245 | 2.91 | 2.13 | 5 |

**Table S3. Structural Connectivity (SCA3＜HC) showing significant difference between SCA3 and HC in the discovery dataset**

| **ROI label** | **ROI label** | **'Mean-SCA3'** | **'Mean-HC'** | **'T-value'** |
| --- | --- | --- | --- | --- |
| 7 | 9 | 11.1 | 13.99 | -3.76 |
| 53 | 57 | 11.51 | 13.36 | -3.64 |
| 56 | 58 | 22.2 | 26.96 | -3.6 |
| 56 | 64 | 6.6 | 7.8 | -3.96 |
| 59 | 67 | 21.23 | 28.84 | -4.08 |
| 60 | 68 | 31.86 | 41.55 | -3.78 |
| 104 | 106 | 9.67 | 11.05 | -3.64 |
| 111 | 119 | 12.69 | 16.35 | -4.85 |
| 113 | 119 | 21.75 | 26.04 | -4.03 |
| 112 | 120 | 12.97 | 15.32 | -3.53 |
| 129 | 131 | 11.34 | 14 | -3.79 |
| 127 | 133 | 11.77 | 14.28 | -3.93 |
| 129 | 133 | 16.31 | 19.47 | -3.77 |
| 135 | 137 | 4.92 | 6.29 | -3.97 |
| 134 | 138 | 10.61 | 13.41 | -3.97 |
| 77 | 143 | 0.07 | 0.09 | -3.23 |
| 137 | 143 | 8.02 | 9.85 | -4.19 |
| 140 | 146 | 11.71 | 13.71 | -3.45 |
| 131 | 149 | 13.31 | 17.28 | -4.12 |
| 127 | 151 | 6.59 | 8.92 | -3.97 |
| 128 | 152 | 5.22 | 6.93 | -3.73 |
| 147 | 153 | 13.6 | 16.5 | -4.06 |
| 149 | 153 | 8.09 | 9.93 | -3.55 |
| 74 | 158 | 3.2 | 4.08 | -3.31 |
| 156 | 160 | 36.61 | 41.69 | -3.87 |
| 159 | 161 | 8.7 | 10.74 | -3.65 |
| 156 | 162 | 4.85 | 6.23 | -3.49 |
| 63 | 163 | 0.13 | 0.17 | -3.65 |
| 27 | 165 | 0.3 | 0.38 | -3.44 |
| 20 | 168 | 0.38 | 0.48 | -3.43 |
| 152 | 176 | 1.64 | 2.22 | -4.48 |
| 127 | 181 | 0.99 | 1.44 | -5.03 |
| 147 | 181 | 1.78 | 2.22 | -3.45 |
| 151 | 181 | 21.88 | 25.07 | -3.97 |
| 152 | 181 | 0.78 | 1.03 | -3.88 |
| 153 | 181 | 10.65 | 12.35 | -3.79 |
| 128 | 182 | 1.18 | 1.69 | -4.84 |
| 68 | 186 | 6.08 | 8.12 | -4.56 |
| 170 | 200 | 0.27 | 0.34 | -3.35 |
| 135 | 201 | 6.37 | 7.82 | -3.97 |
| 143 | 201 | 5.8 | 7.23 | -4.02 |
| 127 | 209 | 16.25 | 19.62 | -3.68 |
| 181 | 209 | 1.15 | 1.49 | -4.2 |
| 199 | 209 | 3.92 | 5.18 | -3.74 |
| 74 | 210 | 0.22 | 0.27 | -3.43 |
| 170 | 210 | 0.31 | 0.38 | -3.5 |
| 9 | 215 | 0.1 | 0.15 | -5.83 |
| 128 | 216 | 0.17 | 0.22 | -4.3 |
| 119 | 217 | 12.42 | 15.09 | -3.66 |
| 106 | 218 | 1.63 | 2 | -3.52 |
| 120 | 218 | 10.6 | 12.98 | -3.79 |
| 210 | 218 | 0.51 | 0.68 | -3.65 |
| 15 | 221 | 0.59 | 0.78 | -3.77 |
| 216 | 222 | 4.36 | 5.58 | -3.52 |
| 157 | 223 | 0.09 | 0.12 | -3.93 |
| 217 | 223 | 1.48 | 1.89 | -4.25 |
| 219 | 223 | 22.36 | 24.96 | -3.43 |
| 128 | 224 | 0.14 | 0.19 | -4.5 |
| 134 | 224 | 0.12 | 0.17 | -4.06 |
| 200 | 224 | 0.23 | 0.31 | -3.85 |
| 210 | 224 | 0.24 | 0.33 | -4.42 |
| 218 | 224 | 4.46 | 5.25 | -3.77 |
| 157 | 225 | 0.08 | 0.1 | -3.35 |
| 219 | 225 | 18.68 | 22 | -4.27 |
| 140 | 226 | 0.04 | 0.05 | -3.41 |
| 160 | 226 | 0.1 | 0.13 | -3.36 |
| 3 | 227 | 2.33 | 3.04 | -3.98 |
| 5 | 227 | 2.16 | 2.75 | -3.63 |
| 15 | 227 | 3.69 | 4.96 | -4.83 |
| 19 | 227 | 2.19 | 2.87 | -4.01 |
| 27 | 227 | 1.06 | 1.35 | -3.36 |
| 53 | 227 | 4.31 | 5.26 | -3.47 |
| 57 | 227 | 2.68 | 3.75 | -5.76 |
| 61 | 227 | 1.61 | 2.14 | -4.08 |
| 63 | 227 | 3.03 | 3.8 | -3.9 |
| 65 | 227 | 1.33 | 1.7 | -4.06 |
| 67 | 227 | 2.32 | 3.02 | -4.76 |
| 151 | 227 | 0.11 | 0.15 | -3.61 |
| 155 | 227 | 1.07 | 1.5 | -4.93 |
| 159 | 227 | 0.77 | 1.07 | -4.68 |
| 161 | 227 | 1.27 | 1.72 | -4.39 |
| 173 | 227 | 3.71 | 5.11 | -4.59 |
| 215 | 227 | 0.86 | 1.48 | -7.35 |
| 221 | 227 | 10.98 | 13.45 | -3.48 |
| 225 | 227 | 4.52 | 5.96 | -5.05 |
| 1 | 228 | 0.71 | 0.93 | -4.01 |
| 4 | 228 | 2.61 | 3.34 | -4.14 |
| 20 | 228 | 4.41 | 5.6 | -4.08 |
| 58 | 228 | 3.48 | 4.54 | -4.97 |
| 128 | 228 | 0.18 | 0.25 | -4.21 |
| 160 | 228 | 1.15 | 1.45 | -3.72 |
| 218 | 228 | 0.57 | 0.78 | -4.66 |
| 220 | 228 | 10.83 | 12.61 | -3.37 |
| 226 | 228 | 8.28 | 10.37 | -4.59 |
| 221 | 229 | 12.68 | 15.05 | -3.69 |
| 225 | 229 | 13.8 | 15.79 | -3.82 |
| 227 | 229 | 12.76 | 16.13 | -4.77 |
| 210 | 230 | 0.37 | 0.47 | -3.49 |
| 216 | 230 | 1.7 | 2.18 | -3.64 |
| 228 | 230 | 13.03 | 16.97 | -5.2 |
| 230 | 232 | 0.68 | 0.88 | -3.74 |
| 62 | 234 | 0.62 | 0.8 | -3.85 |
| 64 | 234 | 0.65 | 0.8 | -3.37 |
| 160 | 234 | 0.29 | 0.38 | -3.53 |
| 228 | 234 | 6.7 | 7.82 | -3.96 |
| 230 | 234 | 1.36 | 1.97 | -5.62 |
| 37 | 235 | 0.05 | 0.06 | -4.49 |
| 53 | 235 | 0.38 | 0.51 | -4.09 |
| 57 | 235 | 1.71 | 2.26 | -3.92 |
| 61 | 235 | 0.11 | 0.16 | -6.16 |
| 63 | 235 | 0.09 | 0.14 | -5.12 |
| 65 | 235 | 0.79 | 0.99 | -3.5 |
| 155 | 235 | 0.5 | 0.67 | -3.84 |
| 159 | 235 | 0.69 | 0.96 | -4.81 |
| 219 | 235 | 0.25 | 0.34 | -4.94 |
| 221 | 235 | 0.58 | 0.9 | -7.53 |
| 225 | 235 | 0.21 | 0.33 | -7.53 |
| 227 | 235 | 2.32 | 3.72 | -8.38 |
| 228 | 235 | 0.09 | 0.11 | -3.82 |
| 62 | 236 | 0.17 | 0.23 | -4.38 |
| 64 | 236 | 0.18 | 0.24 | -3.95 |
| 66 | 236 | 0.8 | 1.06 | -4.47 |
| 68 | 236 | 0.76 | 0.99 | -3.9 |
| 226 | 236 | 0.43 | 0.53 | -3.41 |
| 227 | 236 | 0.12 | 0.15 | -3.8 |
| 228 | 236 | 3.14 | 4.38 | -6.15 |
| 230 | 236 | 0.72 | 0.93 | -4.33 |
| 9 | 237 | 0.32 | 0.43 | -4.14 |
| 57 | 237 | 0.36 | 0.49 | -4.74 |
| 128 | 238 | 0.12 | 0.16 | -4.2 |
| 210 | 238 | 0.13 | 0.17 | -3.32 |
| 237 | 238 | 5.59 | 7.64 | -5.02 |
| 61 | 239 | 0.19 | 0.25 | -4.72 |
| 157 | 239 | 0.16 | 0.21 | -3.77 |
| 223 | 239 | 1.1 | 1.33 | -3.48 |
| 229 | 239 | 0.85 | 1.15 | -4.94 |
| 62 | 240 | 0.27 | 0.35 | -3.59 |
| 216 | 240 | 2.47 | 3.18 | -5.01 |
| 218 | 240 | 3.54 | 4.26 | -3.83 |
| 129 | 241 | 0.18 | 0.23 | -3.55 |
| 223 | 241 | 1.87 | 2.33 | -3.84 |
| 229 | 241 | 0.86 | 1.08 | -3.99 |
| 3 | 243 | 0.33 | 0.42 | -3.48 |
| 9 | 243 | 0.6 | 0.71 | -3.33 |
| 53 | 243 | 0.51 | 0.65 | -3.37 |
| 55 | 243 | 0.46 | 0.57 | -3.35 |
| 57 | 243 | 0.61 | 0.81 | -4.56 |
| 63 | 243 | 0.3 | 0.4 | -4.04 |
| 127 | 243 | 0.24 | 0.3 | -3.35 |
| 129 | 243 | 0.19 | 0.28 | -5.24 |
| 137 | 243 | 0.21 | 0.28 | -3.42 |
| 151 | 243 | 0.31 | 0.41 | -4.25 |
| 155 | 243 | 0.17 | 0.23 | -4.22 |
| 217 | 243 | 4.15 | 5.06 | -3.42 |
| 225 | 243 | 0.82 | 1.02 | -3.68 |
| 228 | 243 | 0.12 | 0.17 | -4.8 |
| 229 | 243 | 1.26 | 1.7 | -4.33 |
| 237 | 243 | 16.16 | 19.99 | -4.19 |
| 8 | 244 | 0.32 | 0.45 | -4.67 |
| 226 | 244 | 1.09 | 1.4 | -3.71 |
| 228 | 244 | 10.07 | 11.85 | -4.33 |
| 234 | 244 | 1.06 | 1.36 | -3.65 |
| 238 | 244 | 15.52 | 19.82 | -4.1 |
| 15 | 245 | 0.29 | 0.37 | -3.33 |
| 61 | 245 | 0.61 | 0.84 | -4.59 |
| 63 | 245 | 0.73 | 0.91 | -3.94 |
| 221 | 245 | 4.63 | 5.91 | -4.64 |
| 227 | 245 | 11.31 | 12.66 | -3.4 |
| 228 | 245 | 0.36 | 0.46 | -3.99 |
| 229 | 245 | 2.28 | 3.68 | -7.13 |
| 235 | 245 | 2.06 | 2.55 | -3.92 |

**Table S4. Functional Connectivity (SCA3＞HC) showing significant difference between SCA3 and HC in the discovery dataset**

| **ROI label** | **ROI label** | **'Mean-SCA3'** | **'Mean-HC'** | **'T-value'** |
| --- | --- | --- | --- | --- |
| 1 | 5 | 0.44 | 0.32 | 3.79 |
| 4 | 26 | 0.6 | 0.49 | 3.6 |
| 15 | 31 | 0.3 | 0.19 | 3.81 |
| 15 | 33 | 0.33 | 0.21 | 4.33 |
| 7 | 53 | 0.49 | 0.38 | 3.82 |
| 17 | 57 | 0.36 | 0.25 | 3.62 |
| 26 | 58 | 0.39 | 0.28 | 3.57 |
| 40 | 58 | 0.26 | 0.15 | 3.84 |
| 25 | 63 | 0.59 | 0.47 | 3.64 |
| 57 | 63 | 0.43 | 0.32 | 3.87 |
| 26 | 64 | 0.51 | 0.4 | 3.61 |
| 58 | 64 | 0.48 | 0.37 | 3.71 |
| 71 | 73 | 0.88 | 0.74 | 3.95 |
| 71 | 75 | 0.7 | 0.58 | 3.48 |
| 73 | 75 | 0.62 | 0.51 | 3.57 |
| 73 | 77 | 0.71 | 0.58 | 3.83 |
| 73 | 79 | 0.62 | 0.49 | 3.64 |
| 74 | 80 | 0.57 | 0.42 | 4.33 |
| 92 | 108 | 0.68 | 0.56 | 3.78 |
| 71 | 127 | 0.38 | 0.27 | 3.73 |
| 73 | 127 | 0.41 | 0.25 | 5.05 |
| 72 | 128 | 0.39 | 0.25 | 4.9 |
| 74 | 128 | 0.37 | 0.24 | 4.36 |
| 76 | 128 | 0.33 | 0.2 | 4.14 |
| 80 | 128 | 0.29 | 0.16 | 4.19 |
| 88 | 128 | 0.3 | 0.17 | 3.96 |
| 124 | 128 | 0.34 | 0.22 | 3.85 |
| 53 | 129 | 0.46 | 0.33 | 4.3 |
| 63 | 129 | 0.64 | 0.5 | 4.87 |
| 129 | 131 | 0.7 | 0.58 | 3.46 |
| 130 | 132 | 0.75 | 0.6 | 3.92 |
| 71 | 133 | 0.42 | 0.26 | 5.39 |
| 73 | 133 | 0.41 | 0.26 | 4.87 |
| 72 | 134 | 0.39 | 0.26 | 4.55 |
| 80 | 134 | 0.32 | 0.18 | 4.72 |
| 88 | 134 | 0.3 | 0.18 | 3.68 |
| 73 | 135 | 0.43 | 0.31 | 4.1 |
| 77 | 135 | 0.34 | 0.22 | 4.28 |
| 81 | 135 | 0.37 | 0.24 | 3.9 |
| 95 | 135 | 0.27 | 0.18 | 3.63 |
| 53 | 137 | 0.29 | 0.18 | 3.57 |
| 127 | 137 | 0.62 | 0.49 | 3.6 |
| 82 | 138 | 0.68 | 0.57 | 3.52 |
| 63 | 139 | 0.71 | 0.59 | 3.6 |
| 54 | 140 | 0.36 | 0.25 | 3.48 |
| 62 | 140 | 0.44 | 0.31 | 3.69 |
| 64 | 140 | 0.72 | 0.6 | 3.53 |
| 74 | 140 | 0.42 | 0.29 | 4.52 |
| 63 | 141 | 0.43 | 0.27 | 4.66 |
| 71 | 141 | 0.38 | 0.25 | 4.33 |
| 73 | 141 | 0.37 | 0.25 | 3.65 |
| 133 | 141 | 0.37 | 0.26 | 3.49 |
| 72 | 142 | 0.34 | 0.22 | 4.4 |
| 74 | 142 | 0.37 | 0.24 | 4.24 |
| 73 | 143 | 0.39 | 0.28 | 3.42 |
| 77 | 143 | 0.41 | 0.31 | 3.48 |
| 97 | 143 | 0.36 | 0.22 | 3.84 |
| 135 | 143 | 0.71 | 0.59 | 3.4 |
| 74 | 144 | 0.36 | 0.26 | 3.34 |
| 73 | 145 | 0.83 | 0.66 | 4.99 |
| 77 | 145 | 0.46 | 0.31 | 4.27 |
| 133 | 145 | 0.42 | 0.3 | 3.77 |
| 72 | 146 | 0.75 | 0.61 | 4.36 |
| 74 | 146 | 0.81 | 0.66 | 4.66 |
| 80 | 146 | 0.4 | 0.25 | 4.7 |
| 128 | 146 | 0.48 | 0.37 | 3.53 |
| 132 | 146 | 0.65 | 0.53 | 3.63 |
| 71 | 147 | 0.33 | 0.21 | 4 |
| 73 | 147 | 0.34 | 0.21 | 4.56 |
| 73 | 148 | 0.35 | 0.23 | 4.38 |
| 74 | 148 | 0.3 | 0.19 | 4.05 |
| 146 | 150 | 0.63 | 0.52 | 3.58 |
| 63 | 155 | 0.51 | 0.38 | 3.9 |
| 129 | 155 | 0.47 | 0.34 | 3.98 |
| 139 | 155 | 0.47 | 0.34 | 3.37 |
| 56 | 156 | 0.71 | 0.57 | 3.95 |
| 64 | 156 | 0.53 | 0.4 | 3.57 |
| 53 | 157 | 0.74 | 0.61 | 3.76 |
| 71 | 157 | 0.71 | 0.61 | 3.6 |
| 129 | 157 | 0.42 | 0.28 | 4.89 |
| 133 | 157 | 0.41 | 0.25 | 4.99 |
| 139 | 157 | 0.48 | 0.36 | 3.54 |
| 155 | 157 | 0.75 | 0.63 | 3.62 |
| 12 | 158 | 0.28 | 0.17 | 3.49 |
| 74 | 158 | 0.79 | 0.66 | 3.45 |
| 140 | 158 | 0.42 | 0.29 | 4.16 |
| 142 | 158 | 0.27 | 0.17 | 3.41 |
| 144 | 158 | 0.29 | 0.17 | 3.5 |
| 53 | 159 | 0.71 | 0.57 | 3.89 |
| 63 | 159 | 0.56 | 0.45 | 3.48 |
| 157 | 159 | 0.6 | 0.46 | 4.23 |
| 63 | 161 | 0.42 | 0.31 | 3.87 |
| 128 | 164 | 0.26 | 0.16 | 3.63 |
| 134 | 164 | 0.28 | 0.16 | 4.13 |
| 142 | 164 | 0.27 | 0.16 | 3.86 |
| 72 | 170 | 0.52 | 0.41 | 3.54 |
| 74 | 170 | 0.63 | 0.51 | 4.09 |
| 128 | 170 | 0.31 | 0.2 | 3.85 |
| 140 | 170 | 0.38 | 0.26 | 4.1 |
| 148 | 170 | 0.27 | 0.16 | 3.88 |
| 63 | 171 | 0.4 | 0.29 | 3.96 |
| 71 | 171 | 0.47 | 0.36 | 3.76 |
| 87 | 171 | 0.22 | 0.12 | 3.87 |
| 159 | 171 | 0.41 | 0.3 | 3.88 |
| 40 | 172 | 0.36 | 0.27 | 3.49 |
| 80 | 172 | 0.32 | 0.21 | 4.06 |
| 86 | 172 | 0.35 | 0.24 | 4.26 |
| 122 | 172 | 0.29 | 0.19 | 3.49 |
| 124 | 172 | 0.35 | 0.25 | 3.6 |
| 140 | 172 | 0.33 | 0.23 | 3.5 |
| 53 | 173 | 0.36 | 0.25 | 3.6 |
| 57 | 173 | 0.26 | 0.17 | 3.4 |
| 124 | 174 | 0.34 | 0.23 | 3.56 |
| 156 | 174 | 0.3 | 0.18 | 3.9 |
| 162 | 174 | 0.3 | 0.19 | 3.59 |
| 11 | 177 | 0.36 | 0.26 | 3.41 |
| 175 | 179 | 0.43 | 0.33 | 3.73 |
| 147 | 181 | 0.64 | 0.53 | 3.59 |
| 175 | 181 | 0.72 | 0.61 | 3.56 |
| 177 | 181 | 0.31 | 0.22 | 3.52 |
| 179 | 181 | 0.4 | 0.28 | 4.75 |
| 147 | 182 | 0.68 | 0.56 | 3.6 |
| 175 | 185 | 0.59 | 0.48 | 3.58 |
| 13 | 187 | 0.83 | 0.71 | 4.04 |
| 88 | 192 | 0.42 | 0.32 | 3.75 |
| 82 | 194 | 0.29 | 0.19 | 4.02 |
| 88 | 194 | 0.41 | 0.29 | 4.25 |
| 106 | 194 | 0.75 | 0.62 | 3.84 |
| 190 | 194 | 0.89 | 0.77 | 3.45 |
| 93 | 195 | 0.25 | 0.17 | 3.41 |
| 103 | 195 | 0.41 | 0.3 | 3.42 |
| 70 | 196 | 0.36 | 0.26 | 3.39 |
| 88 | 196 | 0.39 | 0.28 | 3.78 |
| 194 | 196 | 0.81 | 0.65 | 4.54 |
| 77 | 197 | 0.4 | 0.29 | 4.12 |
| 103 | 197 | 0.36 | 0.26 | 3.44 |
| 70 | 198 | 0.37 | 0.26 | 3.96 |
| 82 | 198 | 0.31 | 0.19 | 4.43 |
| 84 | 198 | 0.38 | 0.27 | 3.9 |
| 88 | 198 | 0.49 | 0.35 | 4.23 |
| 96 | 198 | 0.27 | 0.15 | 5 |
| 104 | 198 | 0.37 | 0.25 | 4.21 |
| 114 | 198 | 0.35 | 0.26 | 3.85 |
| 77 | 199 | 0.35 | 0.24 | 3.71 |
| 74 | 200 | 0.44 | 0.33 | 3.78 |
| 99 | 201 | 0.28 | 0.19 | 3.37 |
| 194 | 206 | 0.74 | 0.58 | 4.41 |
| 196 | 206 | 0.62 | 0.49 | 3.73 |
| 87 | 207 | 0.35 | 0.23 | 4.22 |
| 99 | 207 | 0.25 | 0.14 | 3.76 |
| 74 | 208 | 0.44 | 0.31 | 4.47 |
| 80 | 208 | 0.4 | 0.28 | 4.16 |
| 82 | 208 | 0.24 | 0.11 | 4.76 |
| 88 | 208 | 0.41 | 0.27 | 4.71 |
| 73 | 209 | 0.48 | 0.32 | 5.06 |
| 75 | 209 | 0.42 | 0.26 | 4.54 |
| 77 | 209 | 0.35 | 0.2 | 5.21 |
| 79 | 209 | 0.41 | 0.27 | 4.2 |
| 80 | 209 | 0.34 | 0.23 | 3.42 |
| 81 | 209 | 0.31 | 0.21 | 3.44 |
| 83 | 209 | 0.32 | 0.21 | 3.9 |
| 87 | 209 | 0.36 | 0.22 | 4.62 |
| 99 | 209 | 0.4 | 0.28 | 4.15 |
| 137 | 209 | 0.5 | 0.34 | 4.32 |
| 74 | 210 | 0.45 | 0.3 | 4.78 |
| 76 | 210 | 0.39 | 0.27 | 3.93 |
| 78 | 210 | 0.33 | 0.21 | 3.74 |
| 80 | 210 | 0.37 | 0.23 | 4.17 |
| 82 | 210 | 0.35 | 0.22 | 4.61 |
| 84 | 210 | 0.31 | 0.21 | 3.73 |
| 88 | 210 | 0.4 | 0.23 | 5.17 |
| 96 | 210 | 0.25 | 0.15 | 3.91 |
| 170 | 210 | 0.33 | 0.21 | 3.95 |
| 197 | 210 | 0.59 | 0.47 | 3.49 |
| 208 | 210 | 0.82 | 0.64 | 4.12 |
| 104 | 214 | 0.29 | 0.21 | 3.76 |
| 9 | 215 | 0.29 | 0.19 | 3.45 |
| 191 | 215 | 0.31 | 0.22 | 3.92 |
| 193 | 215 | 0.31 | 0.21 | 3.94 |
| 128 | 216 | 0.27 | 0.17 | 3.62 |
| 208 | 216 | 0.3 | 0.17 | 4.78 |
| 209 | 216 | 0.3 | 0.2 | 3.73 |
| 210 | 216 | 0.34 | 0.22 | 4.42 |
| 128 | 218 | 0.29 | 0.2 | 3.55 |
| 192 | 218 | 0.37 | 0.28 | 3.5 |
| 208 | 218 | 0.33 | 0.23 | 4.58 |
| 209 | 218 | 0.33 | 0.24 | 3.77 |
| 210 | 218 | 0.35 | 0.26 | 4.09 |
| 161 | 221 | 0.21 | 0.11 | 4.1 |
| 161 | 222 | 0.19 | 0.11 | 3.61 |
| 127 | 223 | 0.28 | 0.18 | 3.7 |
| 161 | 223 | 0.29 | 0.19 | 3.97 |
| 2 | 224 | 0.31 | 0.22 | 3.64 |
| 8 | 224 | 0.26 | 0.16 | 4.39 |
| 12 | 224 | 0.31 | 0.21 | 3.66 |
| 18 | 224 | 0.25 | 0.17 | 3.72 |
| 24 | 224 | 0.23 | 0.15 | 3.65 |
| 58 | 224 | 0.28 | 0.2 | 3.48 |
| 60 | 224 | 0.26 | 0.17 | 3.51 |
| 134 | 224 | 0.25 | 0.15 | 4.01 |
| 148 | 224 | 0.32 | 0.21 | 4.25 |
| 160 | 224 | 0.26 | 0.16 | 3.67 |
| 2 | 226 | 0.3 | 0.22 | 3.47 |
| 9 | 226 | 0.27 | 0.19 | 3.39 |
| 10 | 226 | 0.27 | 0.19 | 3.55 |
| 36 | 226 | 0.24 | 0.15 | 3.75 |
| 58 | 226 | 0.18 | 0.1 | 3.3 |
| 60 | 226 | 0.18 | 0.1 | 3.58 |
| 86 | 226 | 0.24 | 0.15 | 3.9 |
| 132 | 226 | 0.18 | 0.09 | 3.84 |
| 146 | 226 | 0.31 | 0.21 | 4.05 |
| 150 | 226 | 0.25 | 0.15 | 4.12 |
| 156 | 226 | 0.16 | 0.08 | 3.33 |
| 158 | 226 | 0.25 | 0.16 | 3.83 |
| 162 | 226 | 0.22 | 0.12 | 4.47 |
| 53 | 229 | 0.37 | 0.21 | 5.45 |
| 55 | 229 | 0.29 | 0.2 | 3.77 |
| 56 | 229 | 0.29 | 0.2 | 3.62 |
| 57 | 229 | 0.31 | 0.19 | 4.36 |
| 67 | 229 | 0.32 | 0.22 | 3.87 |
| 68 | 229 | 0.3 | 0.2 | 3.97 |
| 79 | 229 | 0.27 | 0.18 | 3.47 |
| 127 | 229 | 0.22 | 0.12 | 3.64 |
| 129 | 229 | 0.22 | 0.14 | 3.37 |
| 155 | 229 | 0.31 | 0.17 | 4.97 |
| 159 | 229 | 0.26 | 0.15 | 3.82 |
| 161 | 229 | 0.29 | 0.16 | 5 |
| 201 | 229 | 0.26 | 0.14 | 4.81 |
| 203 | 229 | 0.25 | 0.13 | 4.58 |
| 209 | 229 | 0.25 | 0.16 | 3.74 |
| 28 | 230 | 0.24 | 0.16 | 3.56 |
| 54 | 230 | 0.3 | 0.18 | 4.55 |
| 56 | 230 | 0.33 | 0.24 | 3.44 |
| 58 | 230 | 0.32 | 0.19 | 5.07 |
| 60 | 230 | 0.3 | 0.17 | 4.87 |
| 64 | 230 | 0.35 | 0.27 | 3.62 |
| 68 | 230 | 0.34 | 0.23 | 4.16 |
| 76 | 230 | 0.29 | 0.2 | 3.42 |
| 80 | 230 | 0.3 | 0.19 | 4.72 |
| 82 | 230 | 0.2 | 0.12 | 3.5 |
| 98 | 230 | 0.27 | 0.17 | 3.59 |
| 108 | 230 | 0.3 | 0.21 | 3.41 |
| 124 | 230 | 0.31 | 0.22 | 3.88 |
| 128 | 230 | 0.23 | 0.14 | 3.61 |
| 132 | 230 | 0.25 | 0.15 | 3.69 |
| 134 | 230 | 0.25 | 0.15 | 4.07 |
| 140 | 230 | 0.29 | 0.19 | 3.97 |
| 152 | 230 | 0.27 | 0.18 | 3.45 |
| 156 | 230 | 0.3 | 0.17 | 4.45 |
| 158 | 230 | 0.35 | 0.26 | 3.37 |
| 162 | 230 | 0.31 | 0.18 | 4.78 |
| 202 | 230 | 0.28 | 0.17 | 3.83 |
| 204 | 230 | 0.23 | 0.14 | 3.84 |
| 210 | 230 | 0.28 | 0.18 | 3.97 |
| 5 | 231 | 0.25 | 0.15 | 4.09 |
| 13 | 231 | 0.33 | 0.23 | 4.07 |
| 15 | 231 | 0.35 | 0.26 | 3.67 |
| 43 | 231 | 0.29 | 0.16 | 4.11 |
| 147 | 231 | 0.35 | 0.22 | 4.71 |
| 149 | 231 | 0.32 | 0.22 | 3.72 |
| 6 | 232 | 0.28 | 0.19 | 3.58 |
| 16 | 232 | 0.37 | 0.24 | 5.02 |
| 28 | 232 | 0.31 | 0.22 | 3.75 |
| 148 | 232 | 0.34 | 0.22 | 4.34 |
| 152 | 232 | 0.36 | 0.24 | 4.31 |
| 53 | 235 | 0.26 | 0.17 | 3.63 |
| 57 | 235 | 0.25 | 0.16 | 3.56 |
| 65 | 235 | 0.25 | 0.17 | 3.42 |
| 131 | 235 | 0.22 | 0.14 | 3.7 |
| 8 | 236 | 0.19 | 0.11 | 3.51 |
| 31 | 239 | 0.26 | 0.17 | 4.11 |
| 51 | 239 | 0.3 | 0.21 | 3.6 |
| 107 | 239 | 0.36 | 0.28 | 3.55 |
| 137 | 239 | 0.28 | 0.18 | 3.87 |
| 143 | 239 | 0.33 | 0.22 | 4.15 |
| 147 | 239 | 0.35 | 0.25 | 3.76 |
| 6 | 240 | 0.29 | 0.18 | 4.49 |
| 16 | 240 | 0.31 | 0.22 | 3.62 |
| 20 | 240 | 0.3 | 0.21 | 3.48 |
| 24 | 240 | 0.28 | 0.19 | 3.48 |
| 45 | 241 | 0.31 | 0.23 | 3.47 |
| 127 | 241 | 0.38 | 0.28 | 3.68 |
| 137 | 241 | 0.35 | 0.26 | 3.59 |
| 210 | 241 | 0.36 | 0.26 | 4.14 |
| 12 | 245 | 0.32 | 0.23 | 3.58 |
| 15 | 245 | 0.33 | 0.25 | 3.43 |
| 31 | 245 | 0.26 | 0.17 | 3.73 |
| 41 | 245 | 0.25 | 0.15 | 4.28 |
| 43 | 245 | 0.27 | 0.18 | 3.54 |
| 51 | 245 | 0.32 | 0.23 | 3.45 |
| 53 | 245 | 0.29 | 0.19 | 3.55 |
| 65 | 245 | 0.35 | 0.24 | 4.23 |
| 67 | 245 | 0.31 | 0.21 | 3.64 |
| 129 | 245 | 0.28 | 0.18 | 3.54 |
| 147 | 245 | 0.33 | 0.23 | 3.56 |
| 149 | 245 | 0.35 | 0.25 | 3.65 |
| 150 | 245 | 0.36 | 0.26 | 4.09 |
| 151 | 245 | 0.35 | 0.24 | 3.82 |
| 152 | 245 | 0.35 | 0.26 | 3.65 |
| 161 | 245 | 0.32 | 0.21 | 4.24 |
| 186 | 245 | 0.38 | 0.28 | 3.55 |
| 16 | 246 | 0.32 | 0.23 | 3.35 |
| 20 | 246 | 0.33 | 0.24 | 3.57 |
| 82 | 246 | 0.21 | 0.13 | 3.34 |
