# Supplementary material for: Mapping the Disrupted Connectome in Spinocerebellar Ataxia Type 3: A Network‐Based Statistics Study Identifying Novel Therapeutic Targets for Neuromodulation
Source: CNS Neurosci Ther. 2026 Jul 6;32(7):e71016. doi: 10.1002/cns.71016 (PMC13334373; doi:10.1002/cns.71016)
Supplement: Supplementary file 6 — Data S1: Supporting Information. [file CNS-32-e71016-s006.docx]

**Participants**

**Transcranial magnetic stimulation**

58 participants with SCA3 also underwent MRI, the World Federation of Neurology International Cooperative Ataxia Scale (ICARS) and the Ataxia Assessment and Rating Scale (SARA). Non-motor symptoms were assessed using the Hamilton Depression Rating Scale (HAMD) and the Montreal Cognitive Assessment (MoCA).

Based on previous evidence implicating the paracentral lobule and cerebellar vermis within the cerebello–striato–cortical circuit in the pathophysiology of ataxia[1, 2], the primary motor cortex (M1) and cerebellar vermis were selected as stimulation targets. A combined, dual-site therapeutic protocol was implemented, in which deep TMS (dTMS) applied to the M1 was immediately followed by intermittent theta-burst stimulation (iTBS) targeting the cerebellar vermis. Prior to the first TMS session, the resting motor threshold (RMT) was determined for each patient. In all treatment sessions, stimulation sites were individually identified using surface-based neuro-navigation techniques to ensure the accuracy of this dual-target intervention.

**M1 Stimulation**：Stimulation over the M1 region was delivered using a dTMS protocol with an H7 coil (Shenzhen Yingzhi Technology Co., Ltd., China). The stimulation parameters were as follows: intensity set at 100% RMT, frequency of 10 Hz, 1 pulse per train, train duration of 100 ms, 40 pulses per session, stimulus duration of 4.00 s, intertrain interval of 30 s, and 45 repetitions, yielding a total of 1800 pulses per session. Treatment was administered once daily, 5 days per week, over a 4-week period.

**Cerebellar Vermis Stimulation**：Cerebellar vermis stimulation was applied using an iTBS protocol via a figure-of-eight coil connected to an OSF-6 magnetic stimulator (Wuhan Yiruide Co., China) located in the Department of Radiology of Southwest Hospital. The stimulation intensity was set at 120% RMT. The iTBS parameters consisted of a 50 Hz intra-burst frequency, 3 pulses per burst, intra-burst duration of 0.06 s, and inter-burst frequency of 5 Hz. Each stimulation block contained 10 bursts, lasted 2.00 s, and was followed by an 8 s interval. This cycle was repeated 20 times, resulting in a total of 600 pulses per session. Stimulation was delivered twice daily, with a 5-minute interval between sessions, 5 days per week, for 4 weeks.

References

1. Van Der Horn HJ, Meles SK, Kok JG, et al (2022) A resting-state fMRI pattern of spinocerebellar ataxia type 3 and comparison with 18F-FDG PET. NeuroImage Clin 34:103023. https://doi.org/10.1016/j.nicl.2022.103023

2. Guo J, Jiang Z, Liu X, et al (2023) Cerebello-cerebral resting-state functional connectivity in spinocerebellar ataxia type 3. Hum Brain Mapp 44:927–936. https://doi.org/10.1002/hbm.26113
