# Supplementary material for: Mapping the Disrupted Connectome in Spinocerebellar Ataxia Type 3: A Network‐Based Statistics Study Identifying Novel Therapeutic Targets for Neuromodulation
Source: CNS Neurosci Ther. 2026 Jul 6;32(7):e71016. doi: 10.1002/cns.71016 (PMC13334373; doi:10.1002/cns.71016)
Supplement: Supplementary file 3 — Figure S3: Altered SC in the SCA3 group compared with HCs at an initial threshold of p = 0.0005. (A) Visualization map of NBS‐identified subnetworks with significantly increased SC. In the circular graph, node colors indicate network affiliation. Red edges represent subnetwork 1, and green edges represent subnetwork 2. (B) Visualization of NBS‐identified subnetworks with significantly decreased SC. In the circular graph, node colors indicate network affiliation. Abbreviations: For a complete list of regional node abbreviations and their corresponding full anatomical names, please refer to Table S1. SCA3 = spinocerebellar ataxia type 3; HCs = healthy controls; SC = structural connectivity; NBS = Network‐based statistics. [file CNS-32-e71016-s005.pdf]

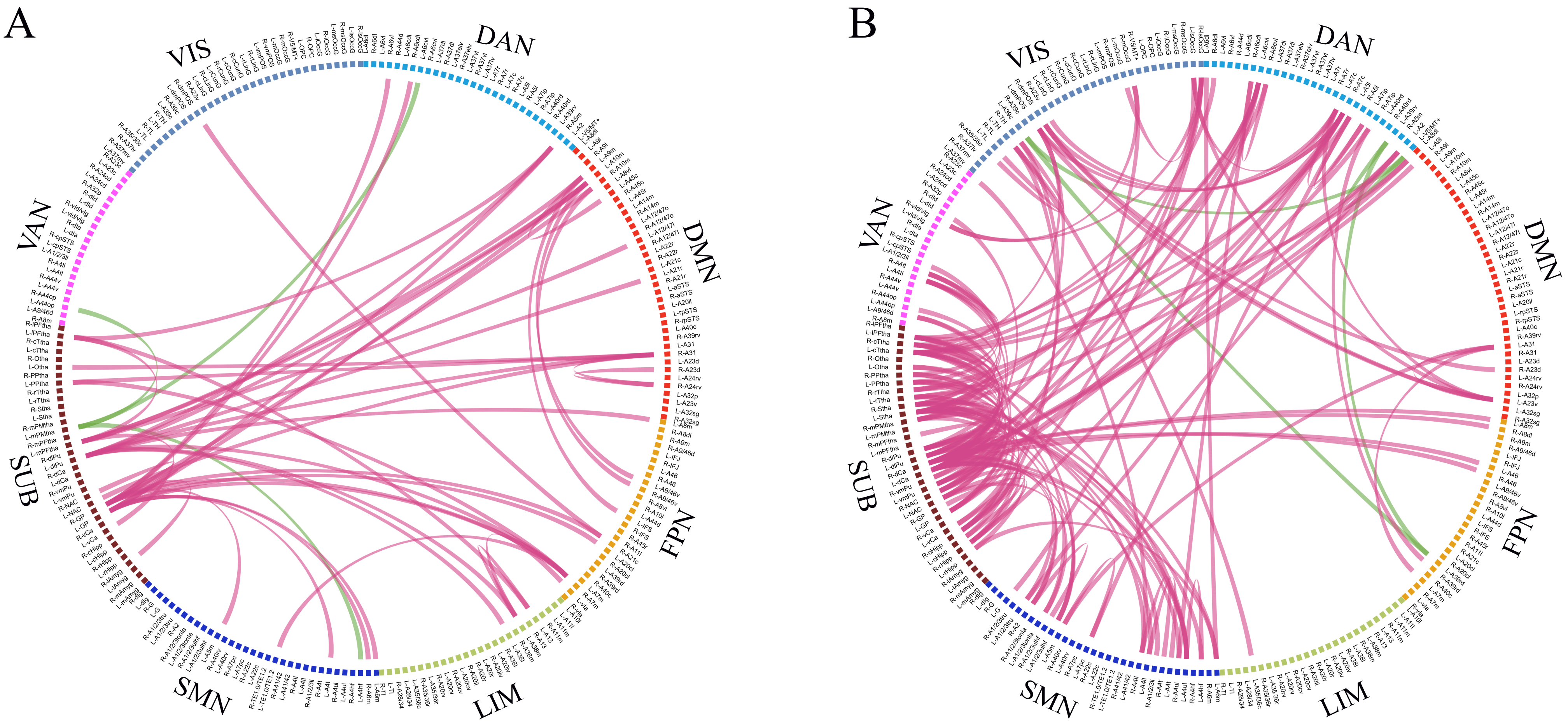
A
VIS
DAN
DMN
FPN
LIM
SMN
SUB
VAN
B
VIS
DAN
DMN
FPN
LIM
SMN
SUB
VAN
